# Physiological and Metabolic Response of *Arthrospira maxima* to Organophosphates

**DOI:** 10.3390/microorganisms10051063

**Published:** 2022-05-21

**Authors:** Amalia Piro, Dante Matteo Nisticò, Daniela Oliva, Francesco Antonio Fagà, Silvia Mazzuca

**Affiliations:** 1 Laboratorio di Biologia e Proteomica Vegetale, Dipartimento di Chimica e Tecnologie Chimiche, Università della Calabria, via P. Bucci 12/C, 87036 Rende, Italy; dante.nistico@unical.it (D.M.N.); daniela.oliva@unical.it (D.O.); silvia.mazzuca@unical.it (S.M.); 2 BIORISI S.r.l.—Oil Fox Europe, Via G. Pinna 78, 88046 Lamezia Terme, Italy; direzione@biorisi.it

**Keywords:** spirulina, *Arthrospira maxima*, glyphosate, proteomics, resistance

## Abstract

The *Spirulina* spp. exhibited an ability to tolerate the organophosphates. This study aimed to explore the effects of the herbicide glyphosate on a selected strain of the cyanobacteria *Arthrospira maxima* cultivated in a company. Experimental cultivations acclimated in aquaria were treated with 0.2 mM glyphosate [N-(phosphonomethyl) glycine]. The culture biomass, the phycocyanin, and the chlorophyll *a* concentrations were evaluated every week during 42 days of treatment. The differentially expressed proteins in the treated cyanobacteria versus the control cultivations were evaluated weekly during 21 days of treatment. Even if the glyphosate treatment negatively affected the biomass and the photosynthetic pigments, it induced resistance in the survival *A. maxima* population. Proteins belonging to the response to osmotic stress and methylation pathways were strongly accumulated in treated cultivation; the response to toxic substances and the negative regulation of transcription seemed to have a role in the resistance. The glyphosate-affected enzyme, chorismate synthase, a key enzyme in the shikimic acid pathway, was accumulated during treatment, suggesting that the surviving strain of *A. maxima* expressed a glyphosate-resistant target enzyme.

## 1. Introduction

Literature of the last ten years has focused on the medium and long-term effects of the persistence of organophosphates, revealing their toxicity and the serious impact on ecosystems of all environmental matrices (air, soil, rivers, sea) and consequently on the health of humans [1,2]. By contrast, related literature also reported conflicting results on the role of organophosphates in perturbing or modifying the activity and microbial composition in soil or water [3,4,5]. Glyphosate is one of the most used organophosphates in industrialized countries; it is a non-selective herbicide with broad-spectrum activity used to eliminate weeds in crops [6]. From the cultivated fields, the glyphosate percolates into the subsoil, thus reaching fresh water and, therefore, the sea, where it causes the global loss of corals and algae [7,8]. Genetically modified crops tolerant to glyphosate were introduced in 1996; however, this has been ineffective in reducing the use of glyphosate and alleviating the severe environmental pollution currently occurring [9]. Therefore, fine-tuning methodologies for the detoxification from these pollutions at a local scale seems to be of great interest for pure and applied research. 

Several studies were devoted to the ability of Cyanobacteria to eliminate glyphosate from the wastewater [10]. Various mechanisms for detoxification among species have been reported: tolerant species seem to be able to use glyphosate as a source of inorganic phosphate, other strains showed a glyphosate metabolism different to those using the phosphonate, and some species were found to possess an insensitive form of glyphosate target enzymes. Particularly *Arthrospira fusiformis* and *Spirulina platensis*, two species belonging to the taxonomic group commonly known as spirulina, showed noteworthy resistance up to 10 mM glyphosate. The authors concluded that the two spirulina species were unable to use glyphosate as a source of phosphorus, suggesting that their tolerance might be due to a very low phosphonate uptake into the cells.

Based on this evidence, this study aims to explore the effects of a deliberate treatment with glyphosate on the selected cultivation of *Arthrospira maxima* (synonymy *Spirulina maxima*), a species largely cultivated as animal and human food additives and as a source of c-phycocyanin, a bioactive antioxidant molecule [11,12].

Here we reported the results on the growth and physiological behavior of the cyanobacteria populations treated with glyphosate at a concentration often found in environmental matrices [2]; the molecular adaptations of *A. maxima* during treatment were investigated by means of a free-label semi-quantitative proteomic approach. The analysis of cyanobacterial proteins has traditionally been carried out using approaches based on electrophoresis [13] and has more recently been applied to *Arthrospira platensis* [14,15]. Genome-wide sequencing of the PCC 8005 strain and its annotation have recently been completed and thus provide key resources to facilitate proteomic approaches to this species [16]. Thousands of proteins have been identified in previous works that have expanded the coverage of the *A. platensis* proteome. Taking advantage of this previous knowledge, we used the one-dimensional gel electrophoresis (SDS-PAGE) combined with LC-MS/MS of both cytosolic and membrane protein fractions to identify proteins differentially accumulated under glyphosate treatment. Despite negative effects on biomass and photosynthetic pigments, proteomics highlighted that modulation of various metabolisms, including the shikimic acid pathway, resulted in resistance to the herbicide of a population of *A. maxima* that survived treatment. 

## 2. Materials and Methods

### 2.1. Arthrospira maxima Culture Preparation

*Arthrospira maxima* strain cultivated according to the Oil Fox^®^ technology and registered trademark Spirulina-fox^®^, covered by the international patent number AR070504B1, were used [17]. Equal volumes of the original cultivation of *A. maxima* were subcultured in 4 aquaria, each containing 20 L of the Zarrouk culture medium [18]. The culture parameters are as follows: pH 10.0 ± 0.1, temperature 27.5 ± 1 °C, and irradiation of 1350 lumens with a light/dark photoperiod of 12 h/12 h of light and oxygen input equal to 800 L/h for each individual aquarium.

### 2.2. A. maxima Treated with Glyphosate (Gly)

Two independent cultivations were treated with 0.2 mM glyphosate (Bayer RASIKAL PRO). All cultivation parameters were kept constant for 42 days of treatment. Immediately after the treatment (t_0_) and every seven days, sampling for biomass analyses, pigment levels, and proteomics was carried out in treated and control cultivations.

### 2.3. Evaluation of the Biomass of Cultivations

*A. maxima* biomass concentration in the cultivations was measured as optical density at a wavelength of 680 nm [19] using a digital Vis-spectrophotometer (Jenway 7310, Cole-Parmer, Staffordshire, UK). In detail, 2 mL of cultivation, taken after vigorous stirring, were transferred in a quartz cuvette, and absorbance was read immediately. Five to seven different samples were taken from the cultivations and measured at each time of treatment.

### 2.4. Analysis of the Concentration of Chlorophyll a and Carotenoids

For the concentrations of chlorophyll *a* and carotenoids, 6 mL of culture, taken after vigorous stirring, were transferred into vials and centrifuged at 13,000 rpm for 5 min to RT. A volume of methanol absolute was added, vortexed, and placed in the dark for 30 min. The samples were then centrifuged at 13,000 rpm for 5 min, and the absorbance of the supernatant was measured at wavelengths of 665 and 652 nm for chlorophyll and 480 nm for the carotenoids using the Vis-spectrophotometer (Jenway 7310, Cole-Parmer, Staffordshire, UK). The concentrations of chlorophyll and carotenoids, expressed as mg/mL, were calculated according to Kumar et al. [20], using the following formulas:

$$\mathrm{Chlorophyll}\;a\;\left(\frac{\mathrm{mg}}{L}\right)=16.29\times OD_{665}-8.54\times OD_{652}$$

$$\mathrm{Carotenoids}\;\left(\frac{\mathrm{mg}}{L}\right)=4\times OD_{480}$$

Five to seven independent extractions of chlorophyll and carotenoids were carried out on the same day for each cultivation. These equations are derived from the difference extinction coefficients, as reported in Porra et al. [21]; therefore, all absorbance measurements at the indicated wavelengths must have the absorbance at 750 nm subtracted. 

### 2.5. Extraction of Phycocyanins from A. maxima Cultivation

The repeatability of the extraction of phycocyanin was evaluated with five repetitions of the extraction on the same day. After vigorous stirring, a volume of cultivation was collected and dried in a vacuum centrifuge at 30 °C. The dried biomass, reduced to a very fine powder, was resuspended in a volume of water at a g/mL ratio, vortexed, and centrifuged at 13,000 rpm for 5 min. The absorbance of the supernatant has been read at 615 nm and 652 nm. The phycocyanin concentration (PC) was finally calculated using the formula reported below [22] and expressed in mg/mL and then in mg/g of dry weight:

$$PC\left(\frac{\mathrm{mg}}{\mathrm{mL}}\right)=\frac{OD_{615}-0.474\times OD_{652}}{5.34}$$

These equations are derived from the different extinction coefficients, as reported in Bennet et al. [22].

### 2.6. Extraction of Proteins from A. maxima Cultivation

The protein extraction was carried out following the acetone/trichloroacetic acid (TCA) precipitation method [23] with some modifications. In total, 150 mL of cultivation were divided into 50 mL falcons and centrifuged at 7900 rpm for 15 min at 4 °C to obtain a pellet of about 1 gr of cyanobacteria.

The pellet was washed with 10 mM Na_2_-EDTA in water and centrifuged at 13,000 rpm for 5 min at RT; the final pellet was frozen in liquid nitrogen and pulverized in a pre-cooled mortar and pestle. The fine powder obtained was divided into several 2 mL Eppendorf, and 10% (*w*/*v*) TCA and 0.07% (*w*/*v*) dithiothreitol (DTT) were added and incubated at −20 °C overnight. After incubation, samples were centrifuged at 13,000 rpm for 20 min at RT; the pellet was washed several times with pre-cooled acetone, 0.07% (*w*/*v*) DTT was added, incubated at −20 °C for 30 min, and centrifuged at 13,000 rpm for 20 min at RT. The pellet was dried for 20 min at RT in a vacuum centrifuge and subsequently solubilized in the loading buffer [24].

Protein concentration was assayed by Bradford’s reagent [25]. A total of 1 μL of the protein extract was added to Bradford’s reagent (1:1; *v*/*v* in water) and incubated in the dark for 5 min.

Absorbance at 595 nm was measured using the Vis-spectrophotometer (Jenway 7310, Cole-Parmer, Staffordshire, UK). Protein concentrations were expressed as mg/mL by using a calibration curve (y = 0.0779x; R_2_ = 0.9719) of the protein standard at different concentrations. Three biological replicates were analyzed for each experimental set. 

### 2.7. Electrophoretic Separation of A. maxima Proteins by SDS-PAGE

Protein samples were separated by SDS-PAGE [24]. Proteins, solubilized in loading buffer, were activated for 4 min at 100 °C. Between 15 μg and 20 μg were loaded onto 12% polyacrylamide/bisacrylamide gels. The electrophoresis was run in a Tris-glycine buffer at 60 mA in the stacking gel and 120 mA in the running gel at 200 V constant power. After electrophoresis, the gels were placed in a Coomassie R-250 solution overnight and then destained through washing in water/ammonium bicarbonate/acetonitrile solution. Digitalized images of the destained SDS-PAGEs were analyzed by the Quantity One 1-D Analysis Software (Bio-Rad, Berkeley, CA, USA) to measure the band densities at each lane of all biological replicates; the amount of protein at bands of 55, 25, and 10 kDa was measured using the marker reference bands at 75, 50, and 25 kDa that contained 150, 750, and 750 ng of proteins, respectively. Each lane of the same SDS-PAGE was divided into six slices from 200 to 10 kDa and manually excised from the gel. Gel slices were processed by in-gel digestion.

### 2.8. Sample Preparation for Mass Analysis:Reduction, Alkylation, and in-Gel Digestion of Proteins

The in-gel digestion procedure required three steps: the reduction, the alkylation, and the tryptic digestion of the polypeptides directly on the gel bands. 

For the reduction step, polypeptide bands were treated with 100 μL of DTT in 50 mM ammonium bicarbonate for 30 min at 56 °C. The reduced polypeptides were alkylated using the ammonium bicarbonate/acetonitrile/iodoacetamide solution. Then trypsin (Promega, Madison WI, USA) dissolved in 25 mM ammonium bicarbonate was added to the reduced-alkylated-polypeptides and incubated at 37 °C overnight [26]. The tryptic digested peptides were extracted by adding the ammonium/bicarbonate 25 mM/acetonitrile/5% formic acid solution; the liquid phase in each sample was extracted, collected in new tubes, and dried in the vacuum centrifuge. Dried samples were treated with 80% formic acid in H_2_O.

### 2.9. Mass Spectrometry Analysis

Twenty microliters of the tryptic peptides were injected into an inverted phase trap column (BioBasicTM LC18 Analytical Column, 300 Å, 5 μm, 50 μm ID × 1 mm long, Thermo Scientific, Sacramento, CA, USA) and separated by an ultra-chromatographic system (UltiMate 3000 RSLC System, Thermo Scientific, Sacramento, CA, USA), at a constant flow of 100 μL/min and with a gradient of 4% of buffer A (2% ACN and 0.1% formic acid in water) to one at 96% of buffer B (2% water and 0.1% formic acid in ACN) for 60 min. The eluting peptides were on-line sprayed in an LTQ XL mass spectrometer (Thermo Scientific, Sacramento, CA, USA). Full scan mass spectra were collected in the linear ion trap in the mass range of *m*/*z* 350 to *m*/*z* 1800 Da, and the 10 most intense precursor ions were selected for collision-induced fragmentation. The acquired MS spectra were used for protein identification.

### 2.10. Bioinformatic Analysis and Proteins Identification of A. maxima

From the MS/MS spectra, protein inference and validation were performed with the Scaffold software 4.8. MS/MS spectra were extracted from raw data by accepting one minimum sequence of three amino acids and fusion scans with the same precursor within one mass window of ±0.4 *m*/*z* over a time interval of ±30 s. The key parameters of research were Scored Peak Intensity, (SPI) ≥ 50%, precursor mass tolerance of ±10 ppm, and mass tolerance of product ions of ±20 ppm. The carbamidomethylation of cysteine was fixed as a modification, and trypsin was selected as the enzyme for the digestion, accepting two missing cleavages per peptide.

Through the X!Tandem software, the proteins found in the samples were carefully aligned and identified in all samples analyzed. Automatic thresholds were used for peptide identification in the software Scaffold. Generally, peptide probabilities are evaluated using a Bayesian approach for the estimation of the local FDR (LFDR) up to a value of 1%. The peptide sequences, using Scaffold 4.8 Q + S system software [27], interfaced with both the database of proteins deduced from generalist protein sequences of Cyanobacteria, that of the genus Arthrospira, deposited in the NCBI database (downloaded on 9 May 2020) and in the bank UniProt data (downloaded on 24 June 2021).

### 2.11. Statistics

Comparison of differences among groups of values for biomass and photosynthetic pigment were analyzed using a *t*-test. All the statistical analyses were performed using XLSTAT (©Addinsoft, Paris, France, released on 2021.3.1.1187) [28]. Significance was defined as *p* ≤ 0.05.

For the proteomics results, a comparison of differences among the groups was carried out using the Differentially Expression and Heat map tools (XLSTAT). The Bonferroni test was used to test the assumption of homogeneity of variances. Threshold for significance was *p* ≤ 0.05. 

The gene enrichment analysis was carried out by BLAST2go software [29] which executes a statistical assessment of differences in functional classes between two groups of sequences based on the Fisher test analysis. By taking a false discovery rate (FDR) significance threshold of 0.05 and a single test *p*-value (Fisher *p*-value), we obtain those functionalities that are strongly significant for proteins in glyphosate-treated samples. The relative frequency of each GO term has been represented for the “biological process” category.

## 3. Results

### 3.1. Dynamics of Arthrospira maxima Cultivation and Biochemical Parameters

**Figure 1:**
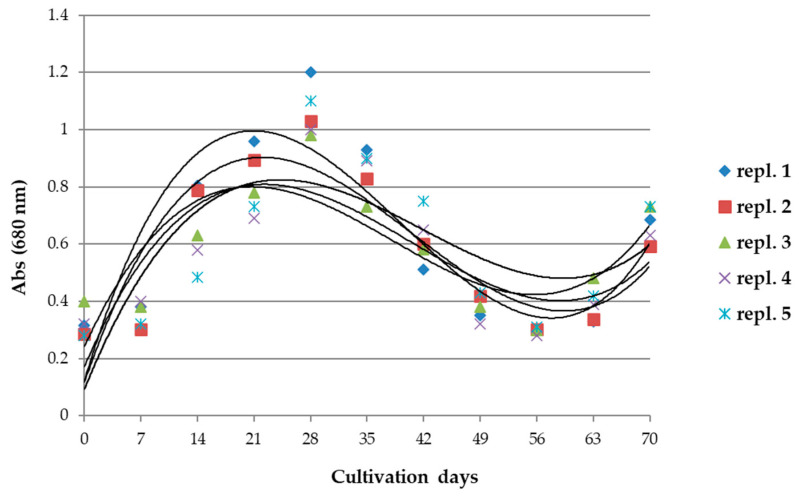
Absorbance values of *A. maxima* samples, taken every 7 days in 2 reference cultivations for a period of 70 days. Growth curves (polynomial of 3rd degree) were calculated for each group of samples. *n* = 5. See Supplementary Table S1A for details.

The biomass and productivity of reference *A. maxima* cultivations were monitored weekly starting from t_0_ up to 70 days of cultivation. In Figure 1, the absorbances of the samples from two reference cultivations are reported. 

As can be seen, maximum cell growth occurred at 21–28 days of cultivation, so growth decreased to its minimum after 56–63 days. At 70 days of cultivation, nutrients had to be added to prevent massive cell mortality. Based on these growth dynamics, we set the reference period for treatments at 42 days of cultivation, in which the concentrations of photosynthetic pigments Chl *a*, phycocyanin, and carotenoids were measured. 

**Figure 2:**
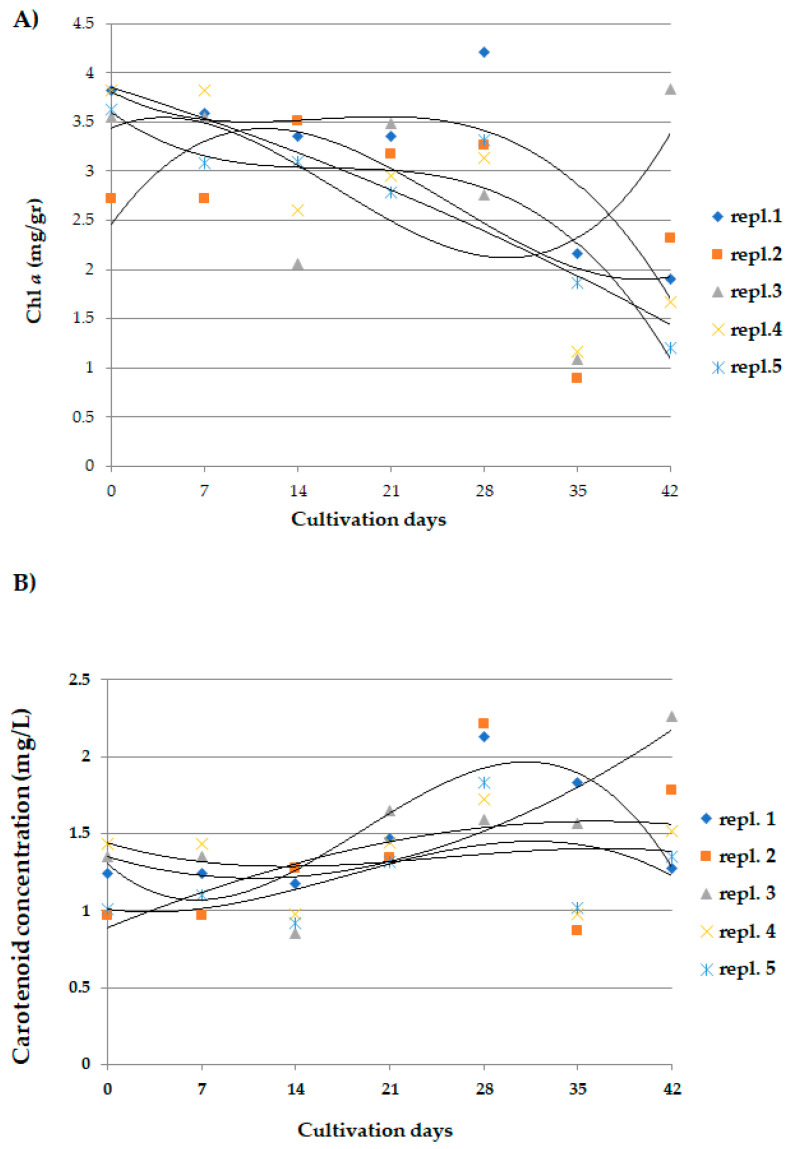
Concentration values of (**A**) Chl *a* and (**B**) total carotenoids in *A. maxima* samples, taken every 7 days in 2 reference cultivations. (**A**) *p* < 0.005 at day 0 and day 35. See Supplementary Table S1B,C for details.

Concentrations of Chl *a* in the two reference cultivations of *A. maxima* varied between samples at the same sampling time; the polynomial regression lines showed a significant decrease (*p* < 0.005) in concentration after the 35th day of culture, with the exception of a single sample which showed the highest value at 42 days (Figure 2A).

The concentrations of total carotenoids had no statistically significant changes among the treated and reference cultivations (Figure 2B).

**Figure 3:**
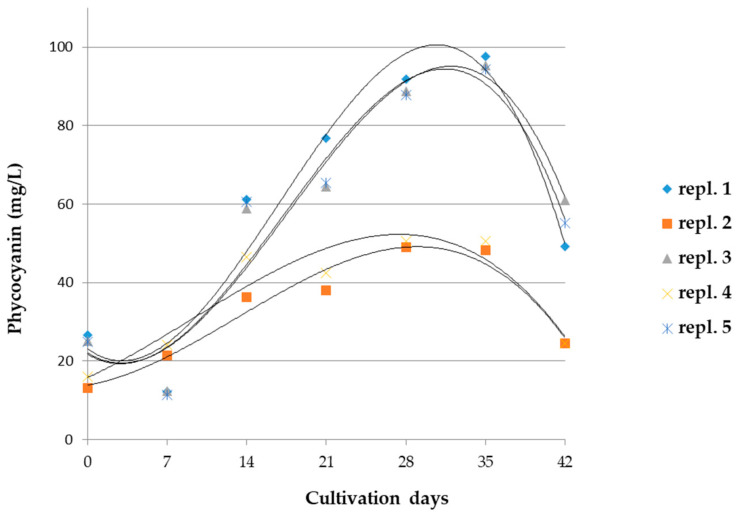
Phycocyanin concentration values in *A. maxima* samples, taken every 7 days in 2 reference cultivations. *n* = 5. Statistical data and parameters are reported in Supplementary Table S1D.

In reference cultivations, the concentrations of phycocyanin increased up to 28–35 days (Figure 3); then at 42 days, it decreased significantly in all analyzed samples (*p* < 0.005).

### 3.2. A. maxima Cultivation Dynamics and Biochemical Parameters during Glyphosate Treatment

**Figure 4:**
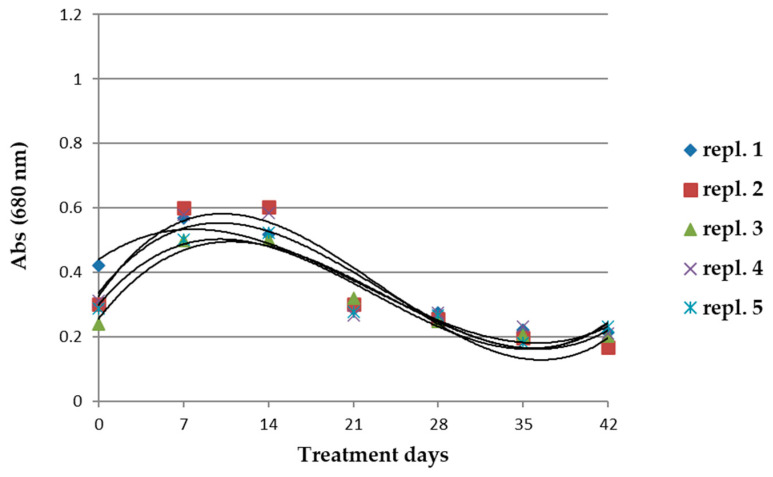
Biomass values evaluated as absorbance of reference samples treated with 0.2 mM glyphosate from cultivations of *A. maxima*. *n* = 5. Details of the data are shown in Supplementary Table S1F.

Figure 4 shows the biomass values in the cultivations during 42 days of treatments with 0.2 mM glyphosate. 

After the treatment with glyphosate, the biomass underwent a significant reduction when compared to the reference cultivations. The maximum biomass occurred after 14 days of treatment, and then it drastically dropped (Figure 4). At the end of the treatment (42 days), the biomass reduced significantly to 50% of that in the reference cultivations (Figure 1). The residual population of *A. maxima* survived; this population was considered to be resistant to the glyphosate treatment. 

**Figure 5:**
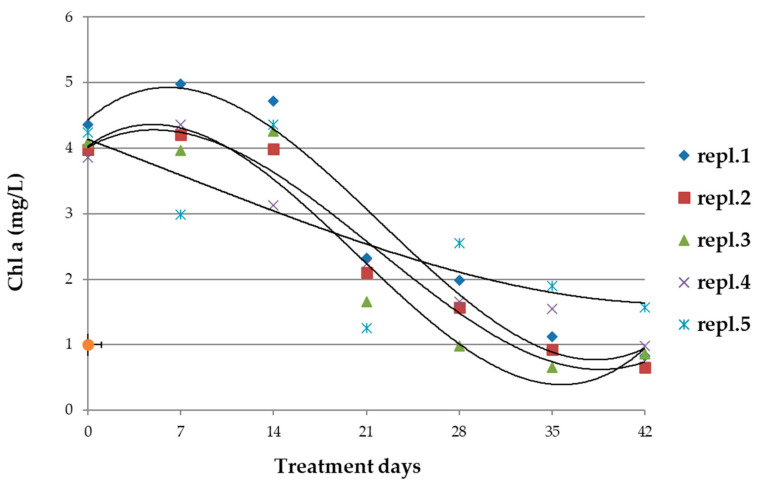
Trend of the concentration of chlorophyll *a* in samples (repl. 1–5) of *A. maxima* cultivations treated with 0.2 mM of glyphosate for 42 days and the mean of the concentration values (±ES) of reference samples. Regression lines (polynomials of 3rd degree) were calculated for each sample group. *n* = 5. The dashed line corresponds to the regression of the values in the reference cultivations. Statistical data and parameters are reported in Supplementary Table S1G.

Treatment with glyphosate induced an increase in chlorophyll concentration during 14 days of treatment (Figure 5); the increase was statistically significant when compared with the values of the reference cultivations in the same period (*p* < 0.05; Supplementary Table S1A). At the end of the treatment, the Chl *a* resulted three times lower than the reference cultivations.

**Figure 6:**
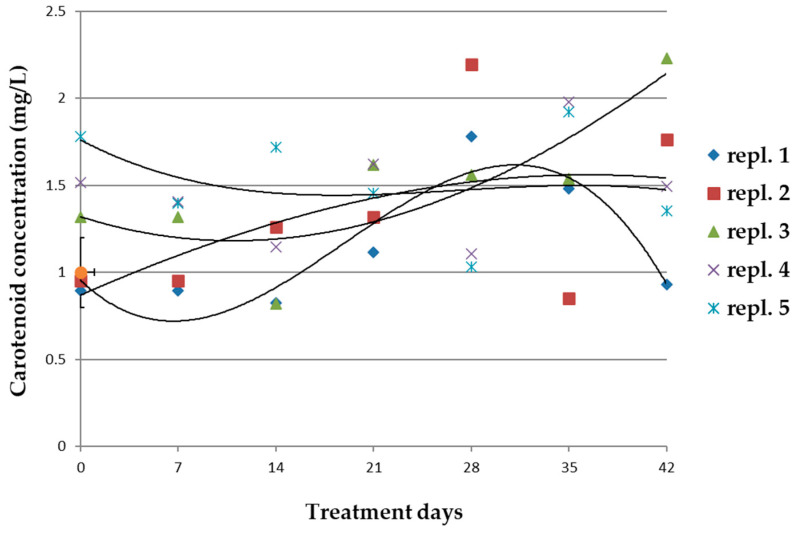
Concentration of carotenoids in biological replicates (*n* = 5) of *A. maxima* cultivations treated with 0.2 mM glyphosate for 42 days. Mean concentration values (±ES) of the reference cultivations and the regression line (dotted line) have been reported. Regression lines were calculated for each sample group. Statistical data and parameters are reported in Supplementary Table S1H.

The concentration of carotenoids was not affected by the treatment; the biological replicates showed variable concentrations at various treatment times, resulting in no significant in comparison with those in the reference cultivations (Figure 6).

**Figure 7:**
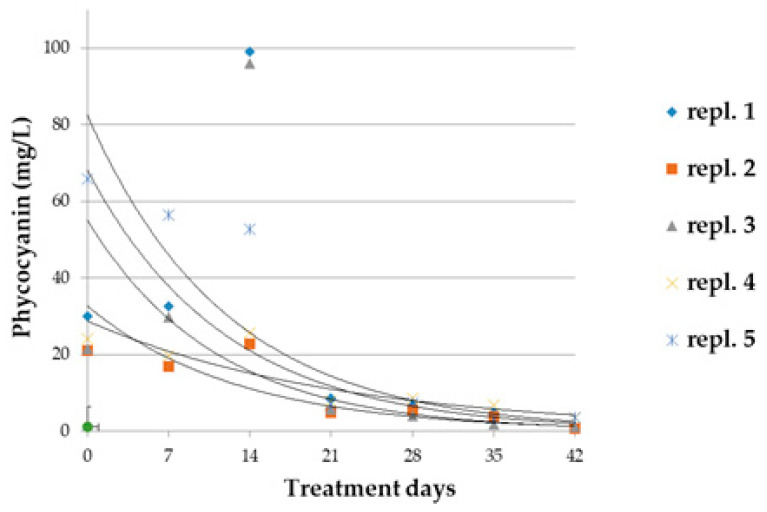
Phycocyanin concentration in biological replicates (*n* = 5) of *A. maxima* cultivations treated with 0.2 mM glyphosate for 42 days. Mean concentration values (±ES) of phycocyanin and the regression line (dotted line) of the reference samples have been also reported. Regression lines were calculated for each group of samples. Statistical data and parameters are reported in Supplementary Table S1I.

Phycocyanin concentrations underwent an exponential reduction in all biological replicates as a result of the treatment (Figure 7). Immediately following the addition of glyphosate to the cultivation (t_0_) and during the following 14 days, the concentrations appeared highly variable among replicates. Then values dropped to zero and, at the end of the treatment, the mean phycocyanin concentration was 1.98 ± 1.26 mg/L, a value 20 times lower than that of the reference cultivations (42.8 ± 14.7 mg/L).

### 3.3. Proteins Differentially Expressed in Cultivations of Arthrospira maxima following Treatment with Glyphosate

Very high yields of purified proteins (more than 60 mg/g fresh weight) have been obtained in reference samples through the extraction protocol optimized in this work. Treatment with glyphosate caused a reduction in the protein content in all samples; after 21 days of treatment, the mean protein concentration of all biological replicates was 0.10 ± 0.03 mg/g fresh weight. 

Samples after 28, 35, and 42 days of treatment yielded tryptic digested peptides, most of which did not receive sequence identification by mass spectrometric analysis (data not shown); therefore, proteomic analysis was performed only in samples up to 21 days of glyphosate treatment.

**Figure 8:**
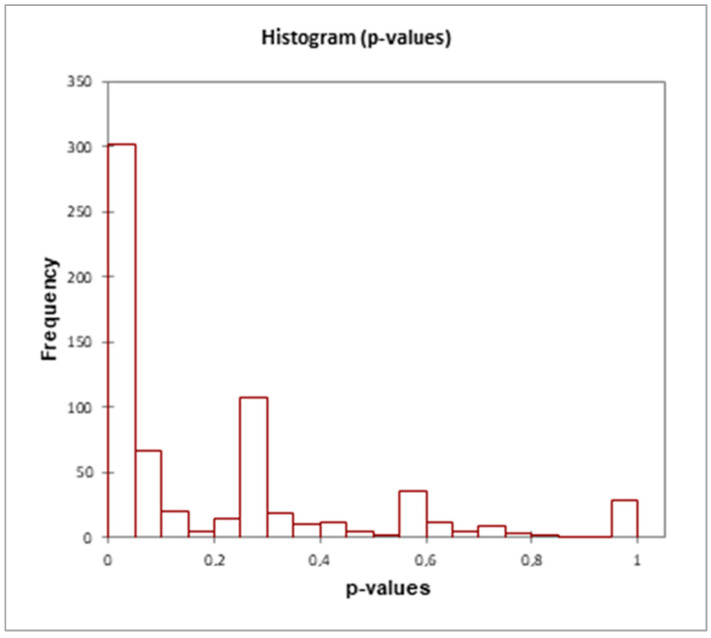
Distribution histograms of differentially expressed proteins in *A. maxima* samples treated with glyphosate compared to control cultivations in the range of *p*-values. XLSTAT 2021.3.1.1187—Differential expression feature. The details of the analysis are shown in Supplementary Tables S2 and S3.

From mass spectrometry and bioinformatics analyses of the protein extracts of *A. maxima* samples, 660 proteins common to both glyphosate-treated and reference samples were identified (see Supplementary Table S2). Semiquantitative analysis, using the spectral counting method, returned differential expression values with different statistical significances depending on the treatment and sampling times. Figure 8 shows the distribution of proteins as a function of their frequency for each range of *p* values; as can be seen, 303 proteins were distributed in a range of very significant *p*-values.

Among these, 143 were differentially expressed with high significance (*** *p* < 0.0001; XLSTAT 2021.3.1.1187—Differential expression feature) in the treated samples compared to the control; another 67 are differentially expressed with *p* = 0.0002. The remainder show differential expression levels with 0.009 < *p* < 0.001 (53 proteins) and with 0.001 < *p* < 0.049 (40 proteins). A total of 357 proteins had no significant changes in their expression following the treatment.

**Figure 9:**
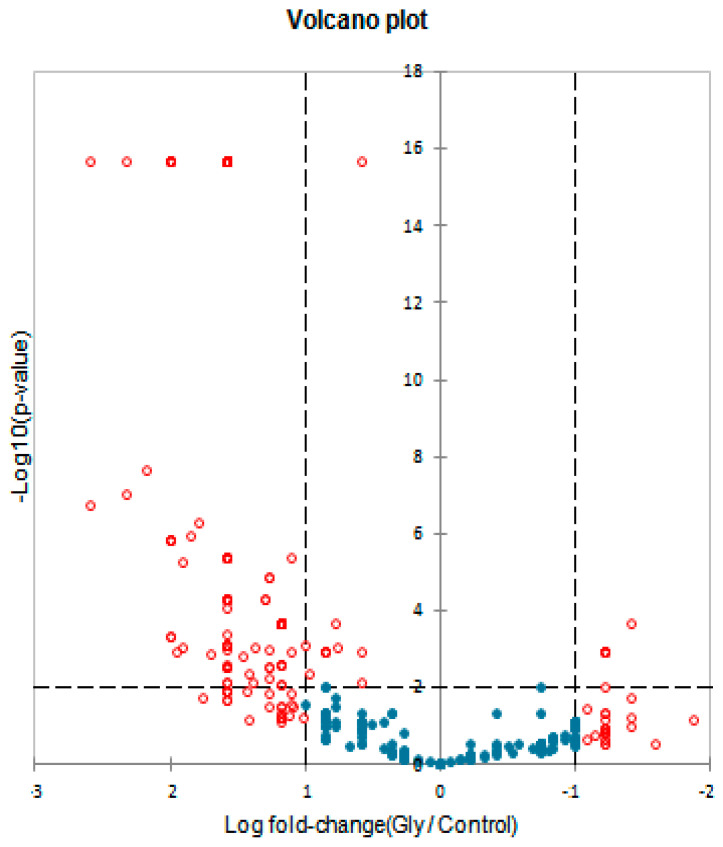
Volcano plot that displays and identifies statistically significant changes in protein expression in *A. maxima* samples treated with 0.2 mM glyphosate compared to the reference samples in terms of change in Log FC (X axis) and *p*-value (Y axis). XLSTAT 2021.3.1.1187- Differential expression feature. The details of the analysis are shown in Supplementary Tables S2 and S3.

The Volcano plot (Figure 9) shows the statistical significance (*p*-values) with respect to the Fold Change (FC) of the differentially expressed proteins in the treated samples compared to the reference samples.

The graph allows us to visually identify proteins that had broadly significant FC that were also statistically significant. The most upregulated proteins were distributed on the left side, the most downregulated proteins were distributed on the right, and the most statistically significant proteins were at the top of the graph. As can be deduced, most of the differentially expressed proteins were accumulated with values up to 2.8 LogFC; furthermore, the expression patterns were very significant for five of these proteins (the dots in the upper left of the graph).

**Figure 10:**
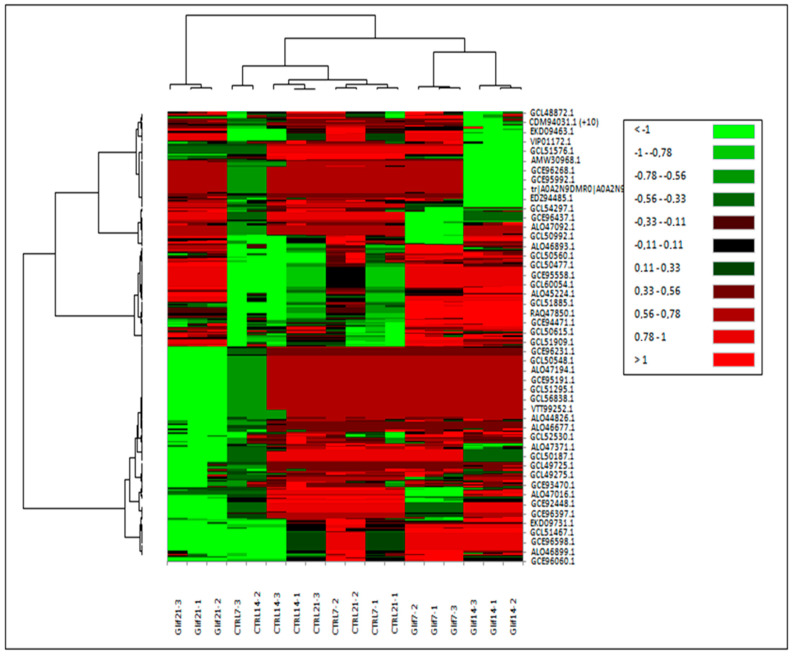
Heat map of proteins differentially expressed in *A. maxima* samples treated with 0.2 mM glyphosate compared to reference samples at various sampling times. Protein expression values were normalized to log2 and cluster analysis was performed using FC levels for proteins with *p* < 0.05. Red indicated a high level of expression; green indicated a low level of expression (XLSTAT 2021.3.1.1187- Heat maps features). The details of the analysis are shown in Supplementary Table S4.

The dynamics of the expression levels of the proteins in the various sampling times during the treatment with glyphosate were evaluated and visualized by means of the Heat Map, as shown in Figure 10.

The glyphosate-treated samples grouped together at various sampling times; this suggested that most of the proteins had the same treatment-induced expression pattern. This was not the case with the reference samples because the 7-, 14-, and 21-day samples clustered together as a result of the expression pattern of proteins that occurred in the reference cultivations.

After 7 days of treatment, the majority of proteins did not vary significantly with respect to the reference samples, and their LogFC values ranged between 0.11 and 0.78. At 14 days of treatment, the percentage of proteins with LogFC < −1 increased, and at 21 days, half of the proteins were under-expressed with values of LogFC < −1. 

**Figure 11:**
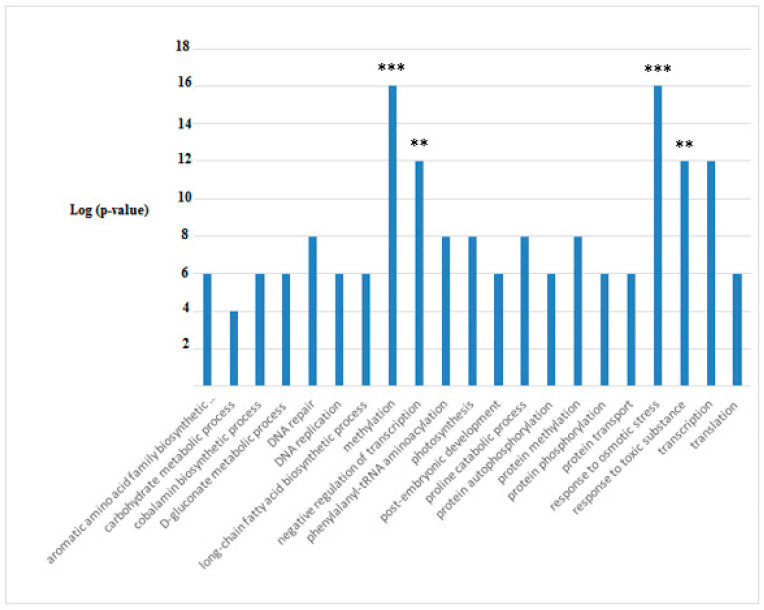
Functional annotation of DAPs in *A. maxima* samples after 21 days of treatment with 0.2 mM glyphosate; the histograms referred to the GO terms to which the enriched proteins, with statistical values of *p* < 0.05, belonged. The data presented are log-transformed *p*-values (corrected with FDR) of GO terms or KEGG (KG) pathways that were found to be enriched in the group of proteins tested. *** *p* < 0.0001; ** *p* < 0.001). The details of the analysis are reported in Supplementary Table S5.

Functional analysis of the DAPs gave significant differences compared to the control only after 21 days of treatment with glyphosate. The Gene Ontology enrichment analysis of the differentially expressed proteins is shown in Figure 11.

As can be seen, the functional analysis revealed very significant changes in the metabolism related to Methylation and to Response to Osmotic Stress (*p* < 0.0001); Response to Toxic Substances and Negative Regulation of Transcription were also significantly changed. All these metabolisms were highly accumulated in glyphosate-treated samples; protein belonging to the remaining metabolisms was reduced in abundance with a significance of *p* < 0.05. 

In Table 1, the Differentially Accumulated Proteins (DAPs) with the lowest *p*-values (*p* < 0.0001) are reported; these proteins were found significantly reduced in abundance following the treatment with glyphosate with respect to the reference cultivations (Table 1).

**Figure 12:**
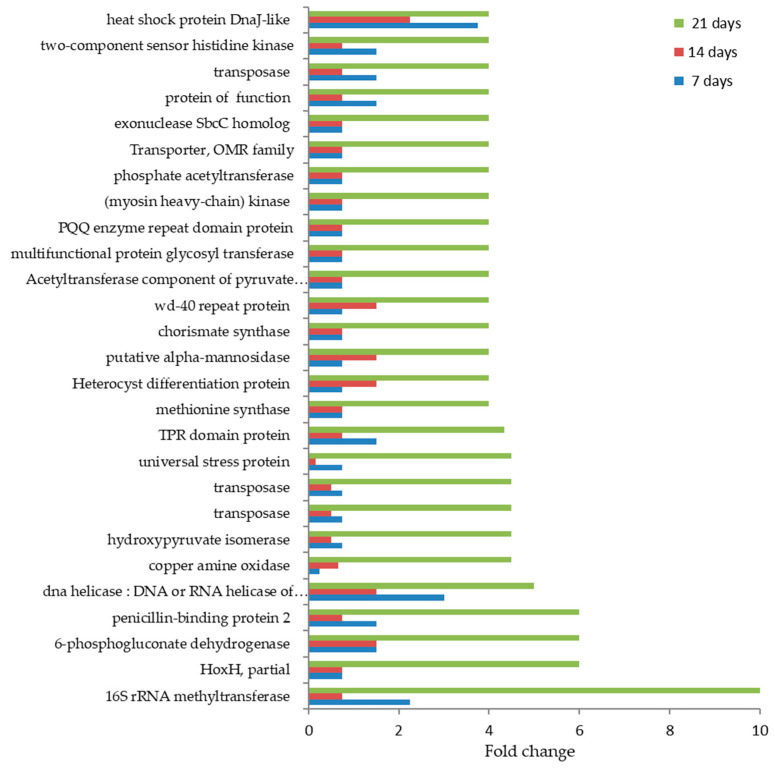
Time course of the highly differentially accumulated proteins during the treatment in treated *A. maxima* cultivation with 0.2 mM glyphosate compared to the reference cultivations. Values are reported as fold changes (Supplementary Table S2).

In Figure 12, the DAPs that were highly accumulated during 21 days of treatment with glyphosate are reported.

Among these, the 16S rRNA methyltransferase was found to be 10-fold enriched with respect to the control; the HoxH protein, the 6-phosphogluconate dehydrogenase, and penicillin-binding protein 2 were 6-fold accumulated. The copper amine hydroxypyruvate, the isomerase oxidase, the universal stress protein, and two transposases were 5-fold accumulated in response to the treatment. Finally, the rest of the highly accumulated proteins varied their abundance of 4-fold respect to the reference samples; among these, the chorismate synthase was worthy to note as these proteins are a key enzyme of the shikimic acid pathway, required for the biosynthesis of all three aromatic amino acids (phenylalanine, tyrosine, and tryptophan). 

## 4. Discussion

Previous studies on glyphosate effects on spirulina species reported a strong tolerance of these microorganisms toward the herbicide [10]. Our results did not confirm for *Arthrospira maxima* a high tolerance to a sublethal dose of glyphosate. However, a survival population of *A. maxima* following the glyphosate treatment has been observed under our conditions. When *A. maxima* cultivations were treated with a 0.2 mM glyphosate, after some days, effects on the sensitive cells occurred. Biomass reduction was the first symptom of the toxic effect of the herbicide on these kinds of cells. Physiological adjustments mediated by an initial increase in chlorophyll *a*, phycocyanin, and by the invariance of carotenoid levels appeared to be part of the resistance mechanisms during the first two weeks of treatment. In particular, we can speculate that the increase in chlorophyll content may be linked to an increase in photosynthesis. It is known that abiotic stresses can induce an increase in light phase reactions to cope with the high energy demand imposed by stressors [30]. After that, strong inhibition of phycocyanin occurred, and the survival strain of *A. maxima* after 42 days of treatment still appeared unable to synthesize large amounts of these molecules.

Proteomic results of the survival population suggested that *A. maxima* responded to glyphosate treatment through the modulation of the osmotic regulation since microorganisms upregulated several proteins belonging to this metabolism; the histidine kinase, enzymes belonging to the proline biosynthesis pathway, the glycolate oxidase, and potassium ion transport were accumulated. It is known that the intracellular accumulation of proline is induced by various stresses also in cyanobacteria [31]; in *Synechocystis* sp., a histidine kinase (Hik33) is induced by both osmotic stress and cold stress. As a response to glyphosate stress, several proteins linked to DNA methylation and post-transductional modification of proteins were upregulated; DNA methylation is linked to epigenetic modifications that are well known as being linked to many important biological processes. In the cyanobacterium model, *Synechocystis* sp. PCC 6803, the variation of “methyloma” (which therefore includes both DNA and protein methylation), has been detected, suggesting that nutritional deficiencies, such as nitrogen, can induce epigenetic modifications that are “inherited” across generations [28]. Here we can hypothesize that glyphosate promoted the methylation processes that involved a reorganization of the “methyloma” of *A. maxima* and a reorganization of the transcription; these data were in agreement with the overexpression of proteins involved in the negative regulation of transcription and with the accumulation of proteins involved in the metabolism of transcription. To complete the picture of the stress response induced by glyphosate, there was the accumulation of proteins belonging to the metabolism of the response to toxic substances, such as the Arsenic resistance protein, the ATP-dependent Clp protease ATP-binding subunit ClpC, and the Cation/multidrug efflux pump.

The SOS response was altered by the treatment; in eukaryotes, the SOS is a global response to DNA damage in which the cell cycle is arrested and DNA repair and mutagenesis are induced [32]. The 16S rRNA methyltransferase was the most highly accumulated protein during the glyphosate treatment and, together with the accumulated PBS lyase heat-like repeat protein and the wd-40 repeat protein, was related to SOS response. In prokaryotes, the SOS response is related to antibiotic resistance and genotoxicity testing under various mutagenic conditions [33,34]. During normal growth, the SOS genes are negatively regulated; accordingly, for the general mechanism for mutagens, we suggested that glyphosate activated SOS genes after DNA damage occurred with the accumulation of single-stranded regions (ssDNA) generated at the replication forks, in which DNA polymerase was blocked. This is consistent with the strong reduction of *A. maxima* growth during the treatment. In this view, the glyphosate exhibited a strong mutagen activity against the cyanobacteria. 

The primary metabolism of gluconeogenesis was also affected by the treatment as the RuBisCo enzyme level was reduced; although there is no evidence in the literature on this effect in cyanobacteria, in higher plants, glyphosate has, in fact, had negative effects on the assimilation of carbon, on the activity of ribulose bisphosphate carboxylase, and the levels of carbohydrate metabolism [35]. On the other hand, the glycolytic process was positively affected to respond to the cell’s high energy demand.

The remarkable tolerance reported in many *spirulina* strains, such as *A. platensis*, a species close to *A. maxima*, was mediated by the ability to degrade glyphosate. High mortality occurred in our conditions suggesting that this was not the case for *A. maxima*. Resistance has been mediated also by the low uptake of organophosphates throughout the cells [10]. In bacteria, it is known that the cell wall acts as a barrier to glyphosate [36]. Interestingly, glyphosate-treated *A. maxima* strongly accumulated the phosphate acetyltransferase, an enzyme involved in the cell wall organization and peptidoglycan biosynthetic process; here we might speculate that reorganization of the cell wall could affect the organophosphate uptake participating in the strategy for resistance. 

In other species, tolerance was obtained by the presence of a resistant form of the glyphosate target enzyme EPSP (5-enolpyruvylshikimate-3-phosphate synthase), a key enzyme of the shikimic acid pathway leading to the aromatic amino acid biosynthesis [10]. It is well known that glyphosate blocks this pathway by inhibiting the EPSPS, which catalyzes the reaction of shikimate-3-phosphate (S3P) and phosphoenolpyruvate to form EPSP [37]. The surviving *A. maxima* population largely accumulated enzymes belonging to the aromatic amino acid biosynthesis, such as the transporter, OMR family, and the chorismate synthase. The first enzyme is a well-characterized aromatic amino acid/H+ symport permease, and the second one catalyzes the 1,4-trans elimination of the phosphate group from 5-enolpyruvylshikimate-3-phosphate (EPSP) to form chorismate which can then be used in phenylalanine, tyrosine, or tryptophan biosynthesis. 

This evidence strongly suggests that *A. maxima* mediated the resistance by the presence of a glyphosate-resistant form of a key enzyme belonging to the shikimate acid pathway, like other cyanobacteria species, but not currently reported for other *spirulina* species. EPSPS gene sequencing in the original culture of *A. maxima* could elucidate which enzyme form it was, whether during the treatment, mutations at the target site of glyphosate occurred, or whether spontaneous strains that expressed the glyphosate-resistant enzyme were already present. 

## 5. Conclusions

By using glyphosate at concentrations found in environmental matrices (0.2 mM), the *A. maxima* residual population showed degrees of resistance to this pollutant. Although culture growth was significantly reduced, a resistant population survived to pollutants after 42 days of treatment. Molecular mechanisms for resistance were the overexpression of metabolisms linked to the response to toxic substances, homeotic stress, oxidative stress, and the reorganization of the cellular “methyloma”. Additionally, the resistance appeared to be mediated by the accumulation of glyphosate-resistant key enzymes belonging to the aromatic amino acid biosynthetic process. 

## Figures and Tables

**Table 1:** Differentially Accumulated Proteins (DAPs) in the Glyphosate treated (Gly) *A. maxima* samples vs. the reference samples after 21 days of treatment. DAPs with the lowest *p*-values and the highest quantitative and qualitative profiles are reported *. The GO- terms, which the DAPs belong to, are also reported.

Protein name and Taxonomy	IDs	*p*-Value	Quantitative Profile ** (Fold Change)		Qualitative Profile	GO Terms
			Reference	Gly		
16S rRNA methyltransferase [*Pseudohongiella spirulinae*]	GCL59050.1	<0.0001	1667 (b)	10 (a)	H.A.	SOS response
HoxH, partial [*Arthrospira platensis* FACHB-440]	EDZ95376.1	<0.0001	1 (b)	6 (a)	H.A.	ADP binding; transferase activity
Penicillin-binding protein 2 [*Arthrospira* sp. PLM2.Bin9]	GCE96397.1	<0.0001	1333 (b)	6 (a)	H.A.	molybdopterin cofactor binding
6-phosphogluconate dehydrogenase [*Arthrospira platensis* NIES-46]	GCE96312.1	<0.0001	1667 (b)	6 (a)	H.A.	glycolytic process
ATP-dependent DNA helicase [*Pseudohongiella spirulinae*]	ALO44826.1	<0.0001	1444 (b)	5 (a)	H.A.	cell division
hypothetical protein NIES46_38410 [*Arthrospira platensis* NIES-46]	ALO47614.1	<0.0001	1 (b)	5 (a)	H.A.	DNA restriction-modification system
Transporter, OMR family [*Pseudohongiella spirulinae*]	GCL52209.1	<0.0001	1 (b)	4 (a)	H.A.	aromatic amino acid family biosynthetic process
Chorismate synthase [*Microcystis aeruginosa* NIES-3804]	GCE92370.1	<0.0001	1 (b)	4 (a)	H.A.	aromatic amino acid family biosynthetic process; chorismate biosynthetic process
hypothetical protein NIES46_48480 [*Arthrospira platensis* NIES-46]	GCL50795.1	<0.0001	1333 (b)	4 (a)	H.A.	ATPase activity; ATP binding; DNA binding
Phosphate acetyltransferase [*Arthrospira platensis* NIES-46]	GCL50871.1	<0.0001	1 (b)	4 (a)	H.A.	cell wall organization; peptidoglycan biosynthetic process; regulation of cell shape
putative DNA helicase [*Microcystis aeruginosa* NIES-3804]	GCL52800.1	<0.0001	1333 (b)	4 (a)	H.A.	DNA metabolism
(myosin heavy-chain) kinase [*Gemmata massiliana*]	GCL49992.1	<0.0001	1 (b)	4 (a)	H.A.	DNA methylation
PBS lyase heat-like repeat protein [*Microcystis aeruginosa* NIES-3804]	GCE96060.1	<0.0001	1667 (b)	4 (a)	H.A.	DNA repair
wd-40 repeat protein: [*Gemmata massiliana*]	GCE92418.1	<0.0001	1333 (b)	4 (a)	H.A.	DNA synthesis involved in DNA repair; double-strand break repair
Acetyltransferase component of pyruvate dehydrogenase complex [*Pseudohongiella spirulinae*]	GCE92614.1	<0.0001	1 (b)	4 (a)	H.A.	glucose metabolic process
Exonuclease SbcC homolog [*Microcystis aeruginosa* NIES-3804]	VTU01960.1	<0.0001	1 (b)	4 (a)	H.A.	integral component of membrane
Putative multifunctional protein glycosyl transferase, tetratricopeptide domains and SAM methyltransferase domains [*Limnospira indica* PCC 8005]	GCE93275.1	<0.0001	1 (b)	4 (a)	H.A.	methylation
PQQ enzyme repeat domain protein [*Gemmataceae bacterium*]	GCE96231.1	<0.0001	1 (b)	4 (a)	H.A.	methylation
Lytic transglycosylase catalytic precursor [*Microcystis aeruginosa* NIES-3804]	GCL46959.1	<0.0001	1333 (b)	4 (a)	H.A.	nitrogen compound metabolic process
Aldolase/epimerase [*Microcystis aeruginosa* NIES-3804]	ALO45161.1	<0.0001	1333 (b)	4 (a)	H.A.	organic acid metabolic process
Transcription-repair coupling factor [*Pseudohongiella spirulinae*]	ALO46521.1	<0.0001	1333 (b)	4 (a)	H.A.	regulation of transcription, DNA-templated
Chromosome segregation protein [*Microcystis aeruginosa* NIES-3804]	GCE95530.1	<0.0001	1333 (b)	4 (a)	H.A.	reproductive process
60 kDa molecular chaperonin 2 [*Microcystis aeruginosa* NIES-3804]	RAQ47071.1	<0.0001	1333 (b)	4 (a)	H.A.	response to stimulus
Transposase [*Microcystis aeruginosa* NIES-3804]	GCE94368.1	<0.0001	1333 (b)	4 (a)	H.A.	signal transduction
hypothetical protein NIES46_27100 [*Arthrospira platensis* NIES-46]	GCL55249.1	<0.0001	1 (b)	4 (a)	H.A.	transmembrane transport
Methionine synthase [*Arthrospira* sp. O9.13F]	ALO46677.1	<0.0001	1 (b)	4 (a)	H.A.	transmembrane transporter activity
hypothetical protein NIES46_44690 [*Arthrospira platensis* NIES-46]	ALO46533.1	<0.0001	1 (b)	4 (a)	H.A.	tricarboxylic acid cycle
hypothetical protein NIES46_24140 [*Arthrospira platensis* NIES-46]	EKD07239.1	<0.0001	1 (b)	4 (a)	H.A.	ubiquitin binding
Two-component sensor histidine kinase [*Microcystis aeruginosa* NIES-3804]	GCL51973.1	<0.0001	1333 (b)	4 (a)	H.A.	viral tail assembly

* the list of all DAPs is reported in Supplementary Table S2; ** Proteins sharing the same letter are not significantly different. a Glyphosate treatment samples, b Reference samples.

## Data Availability

Not applicable.

## Supplementary Materials

The following supporting information can be downloaded at: https://www.mdpi.com/article/10.3390/microorganisms10051063/s1, Table S1: All biochemical data relating to the graphs inserted in the text; Table S2: Differentially expressed proteins identified in *A. maxima* samples in control cultures (CTRL) and cultures treated with 0.2 mM glyphosate (Gly). The UniProt identification codes, the number of mass spectra assigned to each protein at the different sampling times, and the fold change (FC) for each protein for each sample treated with respect to the control at the different sampling times are reported; the Log2 HR expressed as the logarithm of the HR value for each protein. The matrix of values was sorted by LogFC values (gray column) from the most negative (under-expressed in the treatise) to the most positive (over-expressed in the treatise) with respect to the control. Table S3: Statistical analysis of the “Differential expression” in *A. maxima* samples of cultures treated with 0.2 mM glyphosate compared to control samples. Table S4: Heat map of Differentially Accumulated Proteins (DAP) in *A. maxima* samples from cultures treated with 0.2 mM glyphosate versus expression levels of each protein in control samples. Table S5: Gene ontology (GO): biological process, cellular component, molecular function of proteins extracted from *A. maxima* samples of cultures treated with 0.2 mM glyphosate. Table S6: Sequences of identified peptides assigned to each protein. Statistical parameters for each peptide have been reported.
