# Supplementary material for: Physiological and Metabolic Response of Arthrospira maxima to Organophosphates
Source: Microorganisms. 2022 May 21;10(5):1063. doi: 10.3390/microorganisms10051063 (PMC9146548; doi:10.3390/microorganisms10051063)
Supplement: Supplementary file 1 [file microorganisms-10-01063-s001.zip › Supplementary Table S1.pdf]

Supplementary Table S1. All biochemical data relating to the graphs inserted in the text:

A) Absorbance values of *A. maxima* samples taken every 7 days in two separate cultures for a period of 70 days

Biomass Control up to 70 days (Abs 680 nm)							
Days of culture	repl. 1	repl. 2	repl. 3	repl. 4	repl. 5	average control	SD
0	0.315	0.287	0.4	0.32	0.28	0.3204	0.03184
7	0.381	0.301	0.38	0.4	0.32	0.3564	0.03672
14	0.803	0.789	0.63	0.58	0.483	0.657	0.1112
21	0.96	0.893	0.78	0.69	0.73	0.8106	0.09272
28	1.2	1.03	0.981	1	1.1	1.0622	0.07024
35	0.93	0.83	0.732	0.89	0.9	0.8564	0.06032
42	0.51	0.6	0.581	0.65	0.75	0.6182	0.06544
49	0.351	0.42	0.38	0.32	0.43	0.3802	0.03584
56	0.298	0.301	0.3	0.28	0.31	0.2978	0.00712
63	0.328	0.338	0.48	0.39	0.42	0.3912	0.04704
70	0.684	0.593	0.73	0.63	0.73	0.6734	0.04952

B) Concentration values of Chl a in *A. maxima* samples taken every 7 days in two distinct cultures for a period of 42 days

Chlorophyll a (mg/L) Control							
Days of culture	repl. 1	repl. 2	repl. 3	repl. 4	repl. 5	average control	SD
0	3.82093	2.71968	3.55491	3.82093	3.63093	3.509476	0.315918
7	3.58749	2.71968	3.55491	3.82093	3.08749	3.3541	0.360412
14	3.3501	3.51379	2.06048	2.5979	3.101	2.924654	0.476371
21	3.3501	3.17644	3.4858	2.95613	2.785501	3.150794	0.223983
28	4.2146	3.25887	2.75971	3.13359	3.3146	3.336274	0.35133
35	2.16507	0.89836	1.08391	1.15949	1.865	1.434366	0.464535
42	1.90612	2.32195	3.83124	1.67129	1.20612	2.187344	0.711401

C) Total carotenoid concentration values in *A. maxima* samples taken every 7 days in two distinct cultures for a period of 42 days

Carotenoids (mg/L) Control							
Days of culture	repl. 1	repl. 2	repl. 3	repl. 4	repl. 5	average control	SD
0	1.244	0.972	1.348	1.436	1.01	1.202	0.1688
7	1.244	0.972	1.348	1.436	1.1	1.22	0.1472
14	1.176	1.28	0.852	0.976	0.92	1.0408	0.14976
21	1.464	1.34	1.648	1.44	1.32	1.4424	0.09088
28	2.132	2.216	1.588	1.728	1.83	1.8988	0.22016
35	1.832	0.872	1.568	0.98	1.02	1.2544	0.35648
42	1.28	1.784	2.26	1.516	1.351	1.6382	0.30704

D) Phycocyanin concentration values in *A. maxima* samples taken every 7 days in two separate cultures for a period of 42 days

Supplementary Table S1. All biochemical data relating to the graphs inserted in the text:

Phycocyanin (mg/L) Control							
Days of culture	repl. 1	repl. 2	repl. 3	repl. 4	repl. 5	average control	SD
0	26.69738	13.19064	25.00225	15.95094	24.95356	21.15895	5.270532
7	12.24195	21.41386	12.31124	24.1839	11.4236	16.31491	5.187176
14	61.26704	36.2985	58.76667	46.54944	60.63933	52.70419	9.02418
21	76.76442	38.02172	64.54345	42.50861	65.49925	57.46749	13.76186
28	91.90225	48.95543	88.70899	50.5618	87.72397	73.57049	19.0495
35	97.69888	48.16742	95.28502	50.60187	94.3	77.21064	22.26079
42	49.13371	24.45094	61.06255	24.45094	55.17828	42.85528	14.72348

E) Biomass values evaluated as absorbance of control samples

Biomass Control							
Days of culture	repl. 1	repl. 2	repl. 3	repl. 4	repl. 5	average control	SD
0	0.327	0.296	0.395	0.331	0.304	0.3306	0.02592
7	0.374	0.297	0.403	0.396	0.317	0.3574	0.04032
14	0.792	0.757	0.703	0.602	0.508	0.6724	0.09392
21	1.03	0.982	0.752	0.701	0.694	0.8318	0.13936
28	1	1.15	0.932	1	1.1	1.0364	0.07088
35	0.897	0.795	0.727	0.889	0.932	0.848	0.0696
42	0.42	0.4	0.603	0.52	0.68	0.5246	0.09352

F) Biomass values evaluated as absorbance of samples treated with 0.2 mM glyphosate

Biomass Gly							
Treatment days	repl. 1	repl. 2	repl. 3	repl. 4	repl. 5	average	SD
0	0.421	0.302	0.241	0.312	0.289	0.313	0.10664
7	0.568	0.601	0.498	0.578	0.502	0.5494	0.04064
14	0.517	0.603	0.501	0.585	0.523	0.5458	0.08576
21	0.291	0.301	0.321	0.268	0.279	0.292	0.01392
28	0.274	0.256	0.249	0.276	0.267	0.2644	0.01208
35	0.221	0.198	0.207	0.232	0.183	0.2082	0.01096
42	0.214	0.167	0.203	0.212	0.233	0.2058	0.01925

G) Trend of the concentration of chlorophyll a in samples (repl. 1-5) of *A. maxima* cultures treated with 0.2 mM of glyphosate for 42 days and mean of the concentration values (± ES) of control samples

Chlorophyll a (mg/L) Gly									
Treatment days	repl.1	repl.2	repl.3	repl.4	repl.5	average control	SD	average glyphosate	SD
0	4.35451	3.98282	4.08927	3.85741	4.23614	3.509476	0.1688	4.10403	0.153036
7	4.97834	4.21032	3.96214	4.35647	2.98563	3.3541	0.1472	4.09858	0.499756
14	4.72346	3.98564	4.25691	3.12546	4.35698	2.924654	0.14976	4.08969	0.427312
21	2.32652	2.10245	1.65893	2.23659	1.25698	3.150794	0.09088	1.916294	0.366671
28	1.98517	1.56874	0.98546	1.65988	2.54681	3.336274	0.22016	1.749212	0.413422
35	1.12495	0.92561	0.65846	1.54897	1.89546	1.434366	0.35648	1.23069	0.39322
42	0.82556	0.65462	0.85694	0.98546	1.56478	2.187344	0.30704	0.977472	0.238118

Supplementary Table S1. All biochemical data relating to the graphs inserted in the text:

H) Dynamics of the concentration of carotenoids in samples (repl. 1-5) of *A. maxima* cultures treated with 0.2 mM of glyphosate for 42 days and mean of the concentration values (± ES) of control samples

Carotenoids (mg/L) Gly									
**Treatment days**	**repl. 1**	**repl. 2**	**repl. 3**	**repl. 4**	**repl. 5**	**average control**	**SD**	**average glyphosate**	**SD**
0	0.894	0.952	1.318	1.516	1.78	1.142	0.2	1.292	0.2952
7	0.894	0.952	1.318	1.406	1.398	1.166	0.1808	1.1936	0.21648
14	0.826	1.26	0.822	1.146	1.72	0.9868	0.12928	1.1548	0.26816
21	1.114	1.32	1.618	1.623	1.454	1.3884	0.12448	1.4258	0.16704
28	1.782	2.196	1.558	1.108	1.03	1.8448	0.16048	1.5348	0.37264
35	1.482	0.852	1.538	1.98	1.92	1.2004	0.28768	1.5544	0.31648
42	0.93	1.764	2.23	1.496	1.351	1.5842	0.37024	1.5542	0.35424

I) Trend of phycocyanin concentration in samples (repl. 1-5) of *A. maxima* cultures treated with 0.2 mM of glyphosate for 42 days and mean of the concentration values (± ES) of control samples

Phycocyanin (mg/L) Gly									
**Treatment days**	**repl. 1**	**repl. 2**	**repl. 3**	**repl. 4**	**repl. 5**	**average control**	**SD**	**average glyphosate**	**SD**
0	29.98764	21.08277	21.41461	24.03783	65.82397	21.15895	5.270532	32.46936	13.34184
7	32.60412	16.74532	29.64906	19.70037	56.36704	16.31491	5.187176	31.01318	10.77792
14	99.00075	22.65543	96.04569	25.61049	52.71536	52.70419	9.02418	59.20554	30.65414
21	8.630337	4.826592	5.675281	7.781648	7.116105	57.46749	13.76186	6.805993	1.244045
28	6.895131	5.516105	3.940075	8.471161	7.490637	73.57049	19.0495	6.462622	1.387625
35	4.72809	3.743071	1.773034	6.698127	3.370787	77.21064	22.26079	4.062622	1.32039
42	1.083521	0.591011	1.122472	3.546067	3.558052	42.85528	14.72348	1.980225	1.257468
