# Supplementary material for: Physiological and Metabolic Response of Arthrospira maxima to Organophosphates
Source: Microorganisms. 2022 May 21;10(5):1063. doi: 10.3390/microorganisms10051063 (PMC9146548; doi:10.3390/microorganisms10051063)
Supplement: Supplementary file 1 [file microorganisms-10-01063-s001.zip › Supplementary Table S4.pdf]

Supplementary Table S4. Heat map of Differentially Accumulated Proteins (DAP) in *A. maxima* samples from cultures treated with 0.2mM glyphosate versus expression levels of each protein in control samples.

XLSTAT 2021.3.1.1193 - Heat maps - Start time: 30/09/2021 at 15:34:26 / End time: 30/09/2021 at 15:34:31

Features/Individuals table: Workbook = Proteine Glifosato-1.xlsx / Sheet = spectral count / Range = 'spectral count'!$H$3:$Z$663 / 661 rows and 18 columns
Center: Yes
Reduce: Yes
Color scale: Red to green through black
Color calibration: Automatic(Min: -1 / Max: 1)
Seed (random numbers): 1966758294

Summary statistics:

DAP Ids	Observations	Obs. with missing data	Obs. without missing data	Minimum	Maximum	Mean	Std. deviation
AAQ63963.1	18	0	18	1.000	5.000	2.167	1.150
AAX68410.1	18	0	18	1.000	6.000	1.889	1.530
ABF61892.1	18	0	18	1.000	4.000	1.889	1.079
ABV54998.1	18	0	18	0.000	3.000	1.667	0.907
ALO44718.1	18	0	18	1.000	5.000	3.056	1.110
ALO44744.1	18	0	18	1.000	3.000	1.611	0.778
ALO44772.1	18	0	18	1.000	3.000	1.889	0.676
ALO44782.1	18	0	18	1.000	3.000	1.500	0.786
ALO44808.1	18	0	18	1.000	3.000	1.500	0.786
ALO44826.1	18	0	18	1.000	5.000	2.278	1.406
Y1	18	0	18	1.000	4.000	1.944	1.162
ALO44907.1	18	0	18	1.000	3.000	1.500	0.786
ALO44964.1	18	0	18	1.000	3.000	1.944	0.802
ALO44998.1	18	0	18	1.000	4.000	2.111	1.023
ALO45025.1	18	0	18	1.000	3.000	1.500	0.786
ALO45039.1	18	0	18	0.000	5.000	1.500	1.098
ALO45046.1	18	0	18	1.000	3.000	2.111	0.758
ALO45052.1	18	0	18	1.000	4.000	1.500	0.857
ALO45063.1	18	0	18	1.000	3.000	1.889	0.900
ALO45068.1	18	0	18	1.000	4.000	1.667	0.840
ALO45077.1	18	0	18	1.000	3.000	1.667	0.767
ALO45091.1	18	0	18	2.000	6.000	3.722	1.018
ALO45107.1	18	0	18	1.000	3.000	1.500	0.786
ALO45119.1	18	0	18	1.000	3.000	2.278	0.752
ALO45129.1	18	0	18	1.000	3.000	1.500	0.786
ALO45161.1	18	0	18	1.000	4.000	2.056	1.162
ALO45165.1	18	0	18	1.000	3.000	1.556	0.784
ALO45202.1	18	0	18	1.000	4.000	1.500	0.857
ALO45210.1	18	0	18	1.000	3.000	1.889	0.900
ALO45219.1	18	0	18	1.000	4.000	2.278	0.895

ALO45224.1	18	0	18	1.000	4.000	2.278	0.895
ALO45262.1	18	0	18	1.000	3.000	1.500	0.786
ALO45265.1	18	0	18	1.000	4.000	1.611	0.850
ALO45281.1	18	0	18	1.000	3.000	1.500	0.786
ALO45310.1	18	0	18	1.000	3.000	1.833	0.857
ALO45326.1	18	0	18	1.000	3.000	1.889	0.900
ALO45355.1	18	0	18	1.000	3.000	1.889	0.900
ALO45541.1	18	0	18	1.000	3.000	1.667	0.767
ALO45547.1	18	0	18	1.000	3.000	1.667	0.767
ALO45564.1	18	0	18	1.000	4.000	2.111	1.023
ALO45583.1	18	0	18	1.000	3.000	1.500	0.786
ALO45594.1	18	0	18	1.000	3.000	1.500	0.786
ALO45639.1	18	0	18	1.000	5.000	1.500	1.043
ALO45651.1	18	0	18	1.000	4.000	2.444	0.922
ALO45685.1	18	0	18	1.000	4.000	2.056	0.873
ALO45693.1	18	0	18	1.000	4.000	2.556	0.922
ALO45702.1	18	0	18	1.000	3.000	1.500	0.786
ALO45710.1	18	0	18	2.000	5.000	2.944	1.110
ALO45739.1	18	0	18	1.000	3.000	1.500	0.786
ALO45763.1	18	0	18	1.000	4.000	2.278	0.895
ALO45819.1	18	0	18	1.000	4.000	1.944	1.110
ALO45828.1	18	0	18	1.000	4.000	2.111	1.023
ALO45858.1	18	0	18	1.000	3.000	1.889	0.900
ALO45889.1	18	0	18	1.000	3.000	1.500	0.786
ALO45920.1	18	0	18	1.000	3.000	2.056	0.802
ALO45978.1	18	0	18	1.000	4.000	1.944	1.110
ALO46022.1	18	0	18	1.000	3.000	2.222	0.878
ALO46075.1	18	0	18	1.000	3.000	1.500	0.786
ALO46090.1	18	0	18	1.000	6.000	2.944	1.349
ALO46118.1	18	0	18	0.000	4.000	2.111	1.231
ALO46158.1	18	0	18	1.000	3.000	1.667	0.767
ALO46176.1	18	0	18	1.000	3.000	1.500	0.786
ALO46207.1	18	0	18	1.000	3.000	1.667	0.767
ALO46232.1	18	0	18	1.000	5.000	2.389	0.979
ALO46243.1	18	0	18	1.000	4.000	1.500	0.857
ALO46267.1	18	0	18	1.000	3.000	1.500	0.786
ALO46318.1	18	0	18	1.000	3.000	1.444	0.784
ALO46353.1	18	0	18	1.000	3.000	1.444	0.784
ALO46369.1	18	0	18	1.000	4.000	1.944	1.110
ALO46371.1	18	0	18	1.000	5.000	1.611	1.195
ALO46420.1	18	0	18	1.000	3.000	1.444	0.784
ALO46433.1	18	0	18	1.000	3.000	1.444	0.784
ALO46441.1	18	0	18	1.000	4.000	2.278	0.895
ALO46444.1	18	0	18	0.000	4.000	1.778	1.060
ALO46492.1	18	0	18	1.000	5.000	2.611	1.461
ALO46521.1	18	0	18	1.000	4.000	1.778	1.114
ALO46529.1	18	0	18	1.000	4.000	1.944	1.110
ALO46533.1	18	0	18	1.000	4.000	1.611	1.145
ALO46586.1	18	0	18	0.000	4.000	2.056	0.998
ALO46628.1	18	0	18	1.000	4.000	1.444	0.856
ALO46673.1	18	0	18	0.000	4.000	1.444	0.922
ALO46677.1	18	0	18	1.000	5.000	1.611	1.195
ALO46683.1	18	0	18	1.000	4.000	1.944	1.110

ALO46685.1	18	0	18	1.000	3.000	1.444	0.784
ALO46692.1	18	0	18	1.000	3.000	1.444	0.784
ALO46739.1	18	0	18	1.000	4.000	2.278	0.895
ALO46741.1	18	0	18	1.000	3.000	2.222	0.878
ALO46882.1	18	0	18	1.000	3.000	1.889	0.900
ALO46893.1	18	0	18	1.000	4.000	2.111	1.023
ALO46899.1	18	0	18	1.000	4.000	1.889	0.963
ALO46908.1	18	0	18	0.000	6.000	2.500	1.917
ALO46909.1	18	0	18	0.000	4.000	2.111	1.079
ALO46930.1	18	0	18	1.000	3.000	1.444	0.784
ALO46935.1	18	0	18	1.000	3.000	1.444	0.784
ALO47016.1	18	0	18	0.000	3.000	1.611	0.850
ALO47031.1	18	0	18	0.000	4.000	1.389	0.916
ALO47074.1	18	0	18	1.000	3.000	1.444	0.784
ALO47092.1	18	0	18	1.000	3.000	1.444	0.784
ALO47106.1	18	0	18	1.000	4.000	1.444	0.856
ALO47109.1	18	0	18	1.000	3.000	1.611	0.778
ALO47131.1	18	0	18	1.000	5.000	2.389	1.037
ALO47132.1	18	0	18	1.000	3.000	1.611	0.778
ALO47194.1	18	0	18	1.000	3.000	1.444	0.784
ALO47225.1	18	0	18	1.000	4.000	2.278	0.895
ALO47296.1	18	0	18	0.000	5.000	2.222	1.437
ALO47299.1	18	0	18	1.000	5.000	1.833	1.295
ALO47371.1	18	0	18	1.000	4.000	1.778	1.060
ALO47378.1	18	0	18	0.000	3.000	1.444	0.922
ALO47421.1	18	0	18	0.000	7.000	2.389	1.577
ALO47433.1	18	0	18	1.000	6.000	3.333	1.455
ALO47558.1	18	0	18	1.000	3.000	1.889	0.900
ALO47582.1	18	0	18	1.000	3.000	1.611	0.778
ALO47599.1	18	0	18	1.000	3.000	1.611	0.778
ALO47614.1	18	0	18	1.000	5.000	1.778	1.517
ALO47667.1	18	0	18	1.000	3.000	1.889	0.900
AMW26778.1	18	0	18	1.000	3.000	1.611	0.778
AMW26801.1	18	0	18	1.000	3.000	1.444	0.784
AMW27306.1	18	0	18	1.000	3.000	1.889	0.900
AMW27573.1 (+7)	18	0	18	1.000	3.000	1.444	0.784
AMW28495.1	18	0	18	1.000	3.000	1.889	0.900
AMW28500.1 (+8)	18	0	18	0.000	4.000	1.389	0.916
AMW28956.1	18	0	18	0.000	5.000	1.778	1.215
AMW29926.1	18	0	18	1.000	4.000	1.444	0.856
AMW30968.1	18	0	18	1.000	6.000	1.778	1.555
AMW31391.1	18	0	18	0.000	4.000	1.389	0.916
BAI87888.1	18	0	18	1.000	6.000	2.444	1.653
BAI88801.1	18	0	18	0.000	5.000	2.500	1.465
CDM92508.1	18	0	18	1.000	3.000	1.611	0.778
CDM94031.1 (+10)	18	0	18	1.000	4.000	1.444	0.856
CDM95413.1	18	0	18	1.000	3.000	1.611	0.778
CDM95584.1	18	0	18	0.000	5.000	2.333	1.534
CDM96902.1	18	0	18	0.000	5.000	1.889	1.183
CDM96906.1	18	0	18	1.000	3.000	1.444	0.784
EDZ92891.1	18	0	18	1.000	3.000	1.444	0.784
EDZ94485.1	18	0	18	0.000	3.000	1.500	0.924
EDZ95376.1	18	0	18	1.000	6.000	1.944	1.893

EKD05820.1	18	0	18	1.000	4.000	2.056	1.211
EKD05876.1	18	0	18	1.000	4.000	2.056	1.211
EKD06011.1	18	0	18	1.000	3.000	1.611	0.778
EKD06063.1	18	0	18	1.000	3.000	1.889	0.900
EKD06564.1	18	0	18	1.000	4.000	2.056	1.211
EKD07239.1	18	0	18	1.000	4.000	1.611	1.145
EKD07445.1	18	0	18	1.000	4.000	2.056	1.211
EKD07734.1	18	0	18	2.000	4.000	2.722	0.752
EKD08078.1	18	0	18	1.000	5.000	2.611	1.461
EKD08222.1	18	0	18	1.000	3.000	1.611	0.778
EKD08619.1	18	0	18	1.000	4.000	2.389	1.145
EKD08754.1	18	0	18	1.000	3.000	1.444	0.784
EKD09005.1	18	0	18	1.000	3.000	2.389	0.698
EKD09207.1	18	0	18	1.000	4.000	2.056	1.211
EKD09463.1	18	0	18	1.000	3.000	2.056	0.802
EKD09492.1	18	0	18	1.000	4.000	2.111	1.132
EKD09731.1	18	0	18	1.000	3.000	2.222	0.647
EKD09794.1	18	0	18	1.000	5.000	1.944	1.474
EKD09935.1	18	0	18	1.000	4.000	2.889	1.079
EKD10098.1	18	0	18	1.000	3.000	1.611	0.778
EKD10387.1	18	0	18	2.000	5.000	2.944	0.938
EKD10398.1	18	0	18	1.000	3.000	1.444	0.784
EKD10616.1	18	0	18	1.000	4.000	2.389	0.979
EKD11263.1	18	0	18	1.000	4.000	1.444	0.856
EKD11392.1	18	0	18	0.000	5.000	1.444	1.097
EKD11399.1	18	0	18	0.000	4.000	2.056	1.211
EKD11426.1	18	0	18	1.000	3.000	1.611	0.778
EKD11459.1	18	0	18	1.000	3.000	1.889	0.900
EKD11466.1	18	0	18	1.000	3.000	1.444	0.784
EKD11562.1	18	0	18	1.000	4.000	2.222	1.114
EKD11759.1	18	0	18	1.000	5.000	1.778	1.166
GCE92103.1	18	0	18	0.000	7.000	3.000	1.940
GCE92115.1	18	0	18	1.000	4.000	2.389	1.145
GCE92151.1	18	0	18	0.000	7.000	3.778	2.390
GCE92190.1	18	0	18	1.000	3.000	1.611	0.778
GCE92236.1	18	0	18	1.000	5.000	2.778	1.437
GCE92253.1	18	0	18	1.000	3.000	1.444	0.784
GCE92273.1	18	0	18	1.000	4.000	1.944	1.211
GCE92316.1	18	0	18	2.000	5.000	3.056	0.873
GCE92329.1	18	0	18	1.000	3.000	1.444	0.784
GCE92335.1	18	0	18	1.000	6.000	1.778	1.309
GCE92360.1	18	0	18	1.000	6.000	1.778	1.555
GCE92368.1	18	0	18	1.000	3.000	1.444	0.784
GCE92370.1	18	0	18	1.000	5.000	1.611	1.195
GCE92402.1	18	0	18	0.000	4.000	1.556	0.984
GCE92418.1	18	0	18	1.000	4.000	1.778	1.114
GCE92448.1	18	0	18	1.000	3.000	1.611	0.778
GCE92453.1	18	0	18	1.000	3.000	1.889	0.900
GCE92484.1	18	0	18	1.000	3.000	1.444	0.784
GCE92487.1	18	0	18	1.000	3.000	1.444	0.784
GCE92501.1	18	0	18	0.000	5.000	2.833	1.339
GCE92520.1	18	0	18	1.000	3.000	1.444	0.784
GCE92583.1	18	0	18	1.000	3.000	1.444	0.784

GCE92614.1	18	0	18	1.000	4.000	1.611	1.145
GCE92711.1	18	0	18	1.000	3.000	1.444	0.784
GCE92766.1	18	0	18	0.000	5.000	1.389	1.145
GCE92821.1	18	0	18	1.000	3.000	1.611	0.778
GCE92851.1	18	0	18	1.000	3.000	1.611	0.778
GCE92904.1	18	0	18	1.000	3.000	1.444	0.784
GCE92978.1	18	0	18	1.000	4.000	2.222	1.114
GCE92987.1	18	0	18	1.000	3.000	1.444	0.784
GCE92993.1	18	0	18	-1.000	5.000	2.222	1.700
GCE93014.1	18	0	18	0.000	4.000	2.056	1.259
GCE93062.1	18	0	18	0.000	6.000	1.833	1.425
GCE93126.1	18	0	18	0.000	6.000	2.278	1.994
GCE93164.1	18	0	18	1.000	4.000	1.778	1.114
GCE93203.1	18	0	18	1.000	4.000	2.056	1.211
GCE93215.1	18	0	18	1.000	5.000	2.167	1.150
GCE93244.1	18	0	18	1.000	4.000	2.056	1.211
GCE93275.1	18	0	18	1.000	4.000	1.611	1.145
GCE93280.1	18	0	18	1.000	3.000	1.444	0.784
GCE93320.1	18	0	18	0.000	5.000	1.389	1.145
GCE93333.1	18	0	18	1.000	5.000	1.833	1.295
GCE93338.1	18	0	18	0.000	7.000	3.333	1.879
GCE93348.1	18	0	18	1.000	3.000	1.611	0.778
GCE93388.1	18	0	18	1.000	5.000	2.444	1.580
GCE93416.1	18	0	18	1.000	3.000	1.444	0.784
GCE93438.1	18	0	18	1.000	3.000	1.611	0.778
GCE93452.1	18	0	18	0.000	5.000	3.000	1.495
GCE93470.1	18	0	18	0.000	4.000	1.389	0.916
GCE93483.1	18	0	18	0.000	5.000	2.667	1.372
GCE93494.1	18	0	18	0.000	8.000	4.778	2.669
GCE93544.1	18	0	18	1.000	4.000	2.222	1.114
GCE93558.1	18	0	18	1.000	3.000	1.444	0.784
GCE93593.1	18	0	18	1.000	3.000	1.889	0.900
GCE93639.1	18	0	18	0.000	5.000	2.333	1.534
GCE93641.1	18	0	18	1.000	4.000	1.944	1.110
GCE93654.1	18	0	18	0.000	6.000	1.778	1.353
GCE93707.1	18	0	18	1.000	3.000	1.444	0.784
GCE93709.1	18	0	18	1.000	4.000	2.056	1.211
GCE93727.1	18	0	18	1.000	3.000	1.944	0.873
GCE93729.1	18	0	18	0.000	5.000	2.778	1.309
GCE93773.1	18	0	18	1.000	3.000	1.889	0.900
GCE93780.1	18	0	18	1.000	4.000	1.611	0.850
GCE93839.1	18	0	18	1.000	4.000	1.444	0.856
GCE93947.1	18	0	18	1.000	5.000	1.444	1.042
GCE93984.1	18	0	18	0.000	3.000	1.389	0.850
GCE93998.1	18	0	18	0.000	7.000	2.111	1.676
GCE94007.1	18	0	18	1.000	4.000	2.056	0.873
GCE94103.1	18	0	18	1.000	6.000	2.278	1.565
GCE94186.1	18	0	18	1.000	3.000	1.444	0.784
GCE94196.1	18	0	18	1.000	4.000	2.556	0.984
GCE94237.1	18	0	18	1.000	5.000	2.833	1.295
GCE94250.1	18	0	18	1.000	3.000	1.611	0.778
GCE94252.1	18	0	18	1.000	4.000	1.444	0.856
GCE94284.1	18	0	18	1.000	4.000	1.778	1.060

GCE94322.1	18	0	18	1.000	4.000	2.111	0.963
GCE94353.1	18	0	18	1.000	4.000	1.778	0.808
GCE94359.1	18	0	18	1.000	5.000	2.778	1.437
GCE94368.1	18	0	18	1.000	5.000	1.778	1.166
GCE94464.1	18	0	18	1.000	4.000	1.611	0.850
GCE94471.1	18	0	18	1.000	4.000	2.278	1.074
GCE94488.1	18	0	18	1.000	4.000	2.056	1.211
GCE94500.1	18	0	18	1.000	3.000	1.444	0.784
GCE94522.1	18	0	18	0.000	5.000	2.056	1.305
GCE94563.1	18	0	18	1.000	3.000	1.611	0.778
GCE94569.1	18	0	18	1.000	3.000	1.444	0.784
GCE94610.1	18	0	18	1.000	3.000	1.444	0.784
GCE94615.1	18	0	18	2.000	5.000	3.444	0.922
GCE94620.1	18	0	18	1.000	3.000	1.444	0.784
GCE94644.1	18	0	18	1.000	6.000	3.278	1.873
GCE94651.1	18	0	18	1.000	3.000	1.444	0.784
GCE94674.1	18	0	18	1.000	5.000	1.444	1.042
GCE94745.1	18	0	18	1.000	4.000	2.056	1.211
GCE94802.1	18	0	18	1.000	3.000	1.611	0.778
GCE94810.1	18	0	18	1.000	3.000	1.444	0.784
GCE94891.1	18	0	18	0.000	6.000	2.444	1.917
GCE94908.1	18	0	18	1.000	7.000	3.778	1.927
GCE94920.1	18	0	18	1.000	3.000	1.444	0.784
GCE94922.1	18	0	18	1.000	3.000	1.611	0.778
GCE94942.1	18	0	18	1.000	5.000	1.778	1.166
GCE94979.1	18	0	18	1.000	3.000	1.778	0.943
GCE94993.1	18	0	18	1.000	4.000	2.111	1.023
GCE95008.1	18	0	18	0.000	4.000	2.389	1.195
GCE95043.1	18	0	18	0.000	5.000	1.444	1.097
GCE95050.1	18	0	18	1.000	3.000	1.444	0.784
GCE95054.1	18	0	18	1.000	4.000	1.722	0.958
GCE95099.1	18	0	18	1.000	3.000	1.500	0.786
GCE95127.1	18	0	18	1.000	4.000	1.500	0.857
GCE95132.1	18	0	18	0.000	3.000	1.444	0.856
GCE95143.1	18	0	18	0.000	3.000	1.500	0.924
GCE95152.1	18	0	18	1.000	3.000	1.444	0.784
GCE95183.1	18	0	18	1.000	3.000	1.611	0.778
GCE95191.1	18	0	18	1.000	3.000	1.444	0.784
GCE95263.1	18	0	18	1.000	3.000	1.444	0.784
GCE95294.1	18	0	18	1.000	3.000	1.889	0.900
GCE95330.1	18	0	18	1.000	4.000	1.611	1.145
GCE95372.1	18	0	18	1.000	4.000	2.056	1.211
GCE95385.1	18	0	18	1.000	3.000	1.889	0.900
GCE95474.1	18	0	18	1.000	3.000	1.778	0.943
GCE95485.1	18	0	18	1.000	4.000	2.389	0.979
GCE95511.1	18	0	18	1.000	3.000	1.444	0.784
GCE95530.1	18	0	18	1.000	4.000	2.056	1.162
GCE95540.1	18	0	18	1.000	3.000	1.611	0.778
GCE95558.1	18	0	18	1.000	4.000	2.056	1.211
GCE95584.1	18	0	18	1.000	3.000	1.444	0.784
GCE95600.1	18	0	18	1.000	3.000	1.944	0.873
GCE95614.1	18	0	18	1.000	3.000	1.444	0.784
GCE95660.1	18	0	18	1.000	4.000	1.778	1.114

GCE95770.1	18	0	18	1.000	4.000	2.056	1.211
GCE95775.1	18	0	18	1.000	4.000	2.389	0.979
GCE95812.1	18	0	18	1.000	3.000	1.444	0.784
GCE95860.1	18	0	18	1.000	3.000	2.056	0.802
GCE95868.1	18	0	18	1.000	4.000	2.389	0.979
GCE95897.1	18	0	18	1.000	3.000	1.611	0.778
GCE95909.1	18	0	18	1.000	6.000	3.278	1.873
GCE95975.1	18	0	18	1.000	3.000	1.444	0.784
GCE95992.1	18	0	18	1.000	3.000	1.444	0.784
GCE96000.1	18	0	18	1.000	4.000	2.222	1.114
GCE96021.1	18	0	18	1.000	4.000	2.056	1.211
GCE96028.1	18	0	18	1.000	3.000	1.611	0.778
GCE96032.1	18	0	18	1.000	3.000	1.444	0.784
GCE96053.1	18	0	18	1.000	3.000	1.444	0.784
GCE96060.1	18	0	18	1.000	4.000	2.222	1.060
GCE96070.1	18	0	18	1.000	4.000	1.444	0.856
GCE96111.1	18	0	18	1.000	3.000	1.444	0.784
GCE96129.1	18	0	18	1.000	3.000	1.611	0.778
GCE96183.1	18	0	18	1.000	3.000	1.444	0.784
GCE96189.1	18	0	18	1.000	3.000	1.611	0.778
GCE96222.1	18	0	18	1.000	3.000	1.444	0.784
GCE96231.1	18	0	18	1.000	4.000	1.611	1.145
GCE96253.1	18	0	18	1.000	4.000	1.778	1.114
GCE96260.1	18	0	18	1.000	3.000	1.444	0.784
GCE96268.1	18	0	18	1.000	3.000	1.444	0.784
GCE96273.1	18	0	18	1.000	3.000	1.944	0.873
GCE96311.1	18	0	18	1.000	3.000	1.611	0.778
GCE96312.1	18	0	18	1.000	7.000	2.278	1.809
GCE96346.1	18	0	18	1.000	5.000	1.944	1.259
GCE96360.1	18	0	18	1.000	4.000	2.056	1.211
GCE96373.1	18	0	18	1.000	4.000	2.556	0.984
GCE96397.1	18	0	18	1.000	6.000	2.111	1.844
GCE96404.1	18	0	18	1.000	4.000	2.056	1.211
GCE96417.1	18	0	18	1.000	3.000	1.889	0.900
GCE96437.1	18	0	18	1.000	3.000	1.611	0.778
GCE96452.1	18	0	18	1.000	3.000	1.444	0.784
GCE96527.1	18	0	18	1.000	3.000	1.444	0.784
GCE96541.1	18	0	18	1.000	3.000	1.611	0.778
GCE96574.1	18	0	18	1.000	4.000	2.056	1.211
GCE96598.1	18	0	18	1.000	3.000	1.889	0.900
GCE96775.1	18	0	18	1.000	3.000	1.444	0.784
GCE96823.1	18	0	18	1.000	3.000	1.444	0.784
GCE96829.1	18	0	18	1.000	4.000	2.222	1.114
GCE96857.1	18	0	18	1.000	3.000	1.889	0.900
GCL44348.1	18	0	18	1.000	3.000	1.889	0.900
GCL44353.1	18	0	18	1.000	4.000	2.222	1.060
GCL44552.1	18	0	18	1.000	3.000	1.444	0.784
GCL44933.1	18	0	18	1.000	3.000	1.444	0.784
GCL44962.1	18	0	18	1.000	3.000	1.444	0.784
GCL45216.1	18	0	18	1.000	3.000	1.444	0.784
GCL45642.1	18	0	18	1.000	3.000	1.889	0.900
GCL45761.1 (+1)	18	0	18	1.000	3.000	1.444	0.784
GCL46121.1	18	0	18	1.000	3.000	1.444	0.784

GCL46670.1	18	0	18	1.000	3.000	1.611	0.778
GCL46949.1	18	0	18	1.000	3.000	1.444	0.784
GCL46959.1	18	0	18	1.000	4.000	2.056	1.162
GCL47232.1	18	0	18	1.000	3.000	1.944	0.873
GCL48436.1	18	0	18	1.000	4.000	2.056	1.211
GCL48484.1	18	0	18	1.000	4.000	2.056	1.211
GCL48505.1	18	0	18	0.000	4.000	1.444	0.922
GCL48514.1	18	0	18	0.000	3.000	1.500	0.985
GCL48555.1	18	0	18	0.000	3.000	1.611	0.916
GCL48566.1	18	0	18	0.000	5.000	1.556	1.149
GCL48572.1	18	0	18	0.000	4.000	1.444	0.922
GCL48589.1	18	0	18	0.000	5.000	2.444	1.338
GCL48597.1	18	0	18	0.000	4.000	1.611	1.145
GCL48598.1	18	0	18	0.000	4.000	1.389	0.979
GCL48599.1	18	0	18	0.000	6.000	2.611	2.062
GCL48667.1	18	0	18	0.000	5.000	1.556	1.199
GCL48737.1	18	0	18	1.000	4.000	2.444	0.856
GCL48792.1	18	0	18	1.000	4.000	1.444	0.856
GCL48859.1	18	0	18	0.000	5.000	2.444	1.338
GCL48872.1	18	0	18	0.000	5.000	2.111	1.231
GCL48917.1	18	0	18	0.000	4.000	1.389	0.916
GCL48920.1	18	0	18	0.000	5.000	2.333	1.534
GCL48934.1	18	0	18	1.000	5.000	1.444	1.042
GCL48974.1	18	0	18	0.000	5.000	2.000	1.455
GCL49010.1	18	0	18	0.000	5.000	1.444	1.247
GCL49031.1	18	0	18	0.000	5.000	1.444	1.097
GCL49037.1	18	0	18	0.000	5.000	1.500	1.200
GCL49110.1	18	0	18	0.000	4.000	1.944	0.998
GCL49141.1	18	0	18	0.000	5.000	1.389	1.145
GCL49218.1	18	0	18	0.000	5.000	2.500	1.654
GCL49253.1	18	0	18	0.000	6.000	3.333	1.847
GCL49269.1	18	0	18	1.000	5.000	1.611	1.092
GCL49270.1	18	0	18	0.000	5.000	1.944	1.162
GCL49272.1	18	0	18	1.000	5.000	2.111	1.132
GCL49275.1	18	0	18	0.000	5.000	1.444	1.097
GCL49291.1	18	0	18	0.000	6.000	2.056	1.552
GCL49327.1	18	0	18	1.000	4.000	2.278	1.127
GCL49341.1	18	0	18	0.000	4.000	1.389	1.092
GCL49347.1	18	0	18	0.000	5.000	1.778	1.166
GCL49355.1	18	0	18	0.000	4.000	1.389	0.979
GCL49368.1	18	0	18	0.000	4.000	1.389	0.979
GCL49406.1	18	0	18	1.000	5.000	1.611	1.092
GCL49431.1	18	0	18	1.000	4.000	1.611	0.916
GCL49495.1	18	0	18	0.000	4.000	1.944	1.211
GCL49505.1	18	0	18	0.000	4.000	1.444	0.984
GCL49511.1	18	0	18	1.000	5.000	1.889	1.132
GCL49534.1	18	0	18	0.000	4.000	2.111	1.132
GCL49546.1	18	0	18	0.000	4.000	1.778	1.003
GCL49615.1	18	0	18	1.000	3.000	1.444	0.784
GCL49622.1	18	0	18	0.000	4.000	1.500	1.043
GCL49713.1	18	0	18	1.000	4.000	2.389	0.979
GCL49725.1	18	0	18	1.000	4.000	1.444	0.856
GCL49807.1	18	0	18	1.000	4.000	1.778	0.878

GCL49811.1	18	0	18	1.000	3.000	1.444	0.784
GCL49853.1	18	0	18	1.000	4.000	2.056	1.211
GCL49876.1	18	0	18	1.000	5.000	3.056	1.305
GCL49937.1	18	0	18	1.000	3.000	2.056	0.802
GCL49956.1	18	0	18	1.000	5.000	3.500	1.150
GCL49958.1	18	0	18	1.000	4.000	1.889	0.963
GCL49990.1	18	0	18	1.000	4.000	2.222	1.114
GCL49992.1	18	0	18	1.000	4.000	1.611	1.145
GCL50087.1	18	0	18	1.000	3.000	1.889	0.900
GCL50116.1	18	0	18	1.000	3.000	1.889	0.900
GCL50140.1	18	0	18	1.000	3.000	1.611	0.778
GCL50142.1	18	0	18	1.000	3.000	1.611	0.778
GCL50164.1	18	0	18	1.000	3.000	1.611	0.778
GCL50187.1	18	0	18	1.000	3.000	1.611	0.778
GCL50191.1	18	0	18	1.000	3.000	1.889	0.900
GCL50220.1	18	0	18	1.000	4.000	1.778	1.114
GCL50243.1	18	0	18	1.000	3.000	1.889	0.900
GCL50246.1	18	0	18	1.000	4.000	2.056	1.211
GCL50247.1	18	0	18	1.000	3.000	1.889	0.900
GCL50272.1	18	0	18	1.000	3.000	1.611	0.778
GCL50275.1	18	0	18	1.000	3.000	2.056	0.802
GCL50277.1	18	0	18	1.000	3.000	1.611	0.778
GCL50294.1	18	0	18	1.000	4.000	2.222	1.114
GCL50356.1	18	0	18	1.000	3.000	1.444	0.784
GCL50371.1	18	0	18	1.000	3.000	1.889	0.900
GCL50397.1	18	0	18	0.000	6.000	2.444	1.917
GCL50462.1	18	0	18	1.000	4.000	2.278	0.895
GCL50477.1	18	0	18	1.000	4.000	2.222	1.114
GCL50548.1	18	0	18	1.000	3.000	1.444	0.784
GCL50560.1	18	0	18	1.000	5.000	2.333	1.455
GCL50589.1	18	0	18	1.000	4.000	2.722	0.958
GCL50615.1	18	0	18	1.000	5.000	2.167	1.150
GCL50623.1	18	0	18	1.000	4.000	2.056	1.162
GCL50649.1	18	0	18	1.000	7.000	2.389	1.577
GCL50711.1	18	0	18	1.000	3.000	1.444	0.784
GCL50732.1	18	0	18	1.000	4.000	2.056	1.211
GCL50734.1	18	0	18	1.000	4.000	2.722	0.895
GCL50769.1	18	0	18	1.000	3.000	1.611	0.778
GCL50795.1	18	0	18	1.000	4.000	1.778	1.114
GCL50796.1	18	0	18	1.000	5.000	2.167	1.543
GCL50816.1	18	0	18	1.000	3.000	1.611	0.778
GCL50870.1	18	0	18	1.000	3.000	2.111	0.900
GCL50871.1	18	0	18	1.000	5.000	1.611	1.195
GCL50879.1	18	0	18	1.000	3.000	2.056	0.873
GCL50889.1	18	0	18	1.000	4.000	1.444	0.856
GCL50932.1	18	0	18	0.000	4.000	2.111	1.132
GCL50957.1	18	0	18	1.000	3.000	1.444	0.784
GCL50963.1	18	0	18	1.000	4.000	2.556	1.294
GCL50966.1	18	0	18	1.000	3.000	1.444	0.784
GCL50970.1	18	0	18	0.000	5.000	2.333	1.609
GCL50992.1	18	0	18	1.000	3.000	1.889	0.900
GCL51032.1	18	0	18	1.000	4.000	2.056	1.211
GCL51057.1	18	0	18	1.000	3.000	1.611	0.778

GCL51076.1	18	0	18	1.000	3.000	1.444	0.784
GCL51082.1	18	0	18	1.000	4.000	2.056	1.211
GCL51114.1	18	0	18	1.000	3.000	1.444	0.784
GCL51164.1	18	0	18	1.000	3.000	1.444	0.784
GCL51166.1	18	0	18	1.000	3.000	1.889	0.900
GCL51228.1	18	0	18	1.000	3.000	1.611	0.778
GCL51295.1	18	0	18	1.000	3.000	1.444	0.784
GCL51402.1	18	0	18	1.000	3.000	1.444	0.784
GCL51409.1	18	0	18	1.000	3.000	1.889	0.900
GCL51419.1	18	0	18	1.000	3.000	1.611	0.778
GCL51432.1	18	0	18	1.000	7.000	3.778	1.896
GCL51467.1	18	0	18	1.000	3.000	1.889	0.900
GCL51472.1	18	0	18	1.000	3.000	1.778	0.732
GCL51534.1	18	0	18	1.000	3.000	1.444	0.784
GCL51537.1	18	0	18	1.000	3.000	1.889	0.900
GCL51556.1	18	0	18	1.000	3.000	1.444	0.784
GCL51576.1	18	0	18	1.000	3.000	1.611	0.778
GCL51588.1	18	0	18	1.000	3.000	1.611	0.778
GCL51744.1	18	0	18	1.000	4.000	2.056	1.211
GCL51760.1	18	0	18	1.000	3.000	1.611	0.778
GCL51774.1	18	0	18	1.000	3.000	1.444	0.784
GCL51779.1	18	0	18	1.000	3.000	1.444	0.784
GCL51811.1	18	0	18	2.000	11.000	6.778	3.541
GCL51834.1	18	0	18	1.000	3.000	1.444	0.784
GCL51860.1	18	0	18	1.000	3.000	1.444	0.784
GCL51885.1	18	0	18	1.000	4.000	2.389	1.145
GCL51909.1	18	0	18	1.000	4.000	2.111	1.023
GCL51911.1	18	0	18	1.000	4.000	2.222	1.114
GCL51955.1	18	0	18	1.000	5.000	2.500	1.465
GCL51973.1	18	0	18	1.000	4.000	1.778	1.114
GCL51984.1	18	0	18	1.000	4.000	1.611	1.145
GCL51985.1	18	0	18	1.000	3.000	1.444	0.784
GCL52006.1	18	0	18	1.000	3.000	1.444	0.784
GCL52010.1	18	0	18	1.000	3.000	1.444	0.784
GCL52032.1	18	0	18	1.000	3.000	1.444	0.784
GCL52091.1	18	0	18	0.000	3.000	1.389	0.850
GCL52103.1	18	0	18	1.000	3.000	1.889	0.900
GCL52174.1	18	0	18	1.000	3.000	1.611	0.778
GCL52209.1	18	0	18	1.000	4.000	1.611	1.145
GCL52256.1	18	0	18	1.000	5.000	2.056	1.305
GCL52307.1	18	0	18	1.000	4.000	1.778	1.003
GCL52373.1	18	0	18	1.000	3.000	1.889	0.900
GCL52402.1	18	0	18	1.000	3.000	1.444	0.784
GCL52445.1	18	0	18	1.000	3.000	1.611	0.778
GCL52530.1	18	0	18	0.000	3.000	1.556	0.856
GCL52551.1	18	0	18	1.000	4.000	1.611	0.850
GCL52553.1	18	0	18	0.000	3.000	1.389	0.916
GCL52629.1	18	0	18	1.000	3.000	1.778	0.732
GCL52800.1	18	0	18	1.000	4.000	2.056	1.162
GCL52835.1	18	0	18	0.000	3.000	1.389	0.850
GCL52956.1	18	0	18	1.000	3.000	1.444	0.784
GCL52972.1	18	0	18	0.000	4.000	2.000	1.237
GCL53135.1	18	0	18	1.000	4.000	1.778	1.114

GCL53315.1	18	0	18	1.000	3.000	1.611	0.778
GCL53423.1	18	0	18	1.000	3.000	1.778	0.943
GCL53432.1	18	0	18	0.000	6.000	2.333	1.749
GCL54043.1	18	0	18	1.000	3.000	1.444	0.784
GCL54088.1	18	0	18	1.000	3.000	1.889	0.900
GCL54123.1	18	0	18	1.000	3.000	1.444	0.784
GCL54137.1	18	0	18	1.000	3.000	1.444	0.784
GCL54257.1	18	0	18	1.000	4.000	2.556	0.984
GCL54297.1	18	0	18	1.000	5.000	1.444	1.042
GCL54360.1	18	0	18	1.000	4.000	2.222	1.114
GCL54440.1	18	0	18	1.000	3.000	1.611	0.778
GCL54469.1	18	0	18	1.000	3.000	1.444	0.784
GCL54487.1	18	0	18	1.000	3.000	1.444	0.784
GCL54821.1	18	0	18	1.000	3.000	1.611	0.778
GCL54996.1 (+1)	18	0	18	1.000	5.000	1.778	1.517
GCL55041.1	18	0	18	1.000	3.000	1.444	0.784
GCL55246.1	18	0	18	2.000	4.000	2.722	0.752
GCL55249.1	18	0	18	1.000	4.000	1.611	1.145
GCL55361.1	18	0	18	1.000	6.000	1.611	1.335
GCL55369.1	18	0	18	1.000	5.000	2.333	1.455
GCL55509.1	18	0	18	1.000	3.000	1.500	0.786
GCL55544.1	18	0	18	1.000	4.000	2.056	1.211
GCL55554.1	18	0	18	1.000	3.000	1.611	0.778
GCL55567.1	18	0	18	1.000	4.000	1.444	0.856
GCL55691.1	18	0	18	1.000	4.000	2.389	0.979
GCL55791.1	18	0	18	1.000	3.000	1.500	0.786
GCL55796.1	18	0	18	1.000	3.000	1.444	0.784
GCL55834.1	18	0	18	1.000	3.000	1.444	0.784
GCL55919.1	18	0	18	1.000	4.000	2.222	1.114
GCL55921.1	18	0	18	1.000	4.000	1.889	1.023
GCL55992.1	18	0	18	1.000	4.000	1.944	1.110
GCL56109.1	18	0	18	1.000	3.000	1.444	0.784
GCL56355.1	18	0	18	1.000	4.000	1.944	1.056
GCL56477.1	18	0	18	1.000	4.000	3.056	1.162
GCL56478.1	18	0	18	1.000	6.000	2.611	1.650
GCL56723.1	18	0	18	1.000	3.000	1.778	0.732
GCL56838.1	18	0	18	1.000	3.000	1.444	0.784
GCL57440.1	18	0	18	1.000	3.000	1.611	0.778
GCL57442.1	18	0	18	1.000	3.000	1.889	0.900
GCL58385.1	18	0	18	1.000	3.000	1.611	0.778
GCL58853.1	18	0	18	1.000	3.000	1.444	0.784
GCL58939.1	18	0	18	1.000	4.000	2.222	1.114
GCL59014.1	18	0	18	1.000	3.000	1.444	0.784
GCL59050.1	18	0	18	1.000	11.000	2.944	3.351
GCL59051.1	18	0	18	1.000	3.000	1.611	0.778
GCL59281.1	18	0	18	1.000	3.000	2.056	0.802
GCL59779.1	18	0	18	1.000	7.000	2.111	2.272
GCL59897.1	18	0	18	1.000	3.000	1.444	0.784
GCL60054.1	18	0	18	1.000	4.000	2.056	1.211
GCL60156.1	18	0	18	1.000	4.000	2.389	0.979
GCL60619.1	18	0	18	1.000	3.000	1.889	0.900
GCL60907.1	18	0	18	1.000	4.000	2.222	1.114
RAQ38716.1	18	0	18	1.000	3.000	1.444	0.784

RAQ38817.1	18	0	18	1.000	3.000	1.611	0.778
RAQ38879.1	18	0	18	1.000	4.000	2.056	1.211
RAQ38927.1	18	0	18	1.000	4.000	1.778	1.114
RAQ39425.1	18	0	18	1.000	4.000	2.111	1.023
RAQ39445.1	18	0	18	1.000	3.000	1.611	0.778
RAQ39680.1	18	0	18	1.000	3.000	1.611	0.778
RAQ39932.1	18	0	18	0.000	3.000	1.389	0.850
RAQ40164.1	18	0	18	1.000	3.000	1.611	0.778
RAQ40448.1	18	0	18	1.000	3.000	1.444	0.784
RAQ40522.1	18	0	18	1.000	4.000	1.444	0.856
RAQ40775.1	18	0	18	1.000	3.000	1.889	0.900
RAQ41462.1	18	0	18	1.000	4.000	2.556	1.294
RAQ42577.1	18	0	18	1.000	3.000	1.444	0.784
RAQ42588.1	18	0	18	1.000	3.000	1.444	0.784
RAQ43317.1	18	0	18	1.000	4.000	2.222	1.114
RAQ43332.1	18	0	18	1.000	3.000	1.444	0.784
RAQ44173.1	18	0	18	1.000	4.000	3.056	1.162
RAQ45356.1	18	0	18	1.000	3.000	1.611	0.778
RAQ45823.1	18	0	18	1.000	3.000	1.444	0.784
RAQ45915.1	18	0	18	1.000	3.000	1.444	0.784
RAQ46741.1	18	0	18	1.000	4.000	2.222	0.732
RAQ46791.1	18	0	18	1.000	4.000	2.722	1.074
RAQ47004.1	18	0	18	1.000	3.000	1.444	0.784
RAQ47071.1	18	0	18	1.000	4.000	2.056	1.162
RAQ47232.1	18	0	18	1.000	4.000	2.222	1.114
RAQ47549.1	18	0	18	2.000	5.000	3.056	1.162
RAQ47585.1	18	0	18	1.000	3.000	1.944	0.873
RAQ47850.1	18	0	18	1.000	4.000	2.222	1.114
RAQ48331.1	18	0	18	1.000	3.000	2.222	0.878
RAQ48378.1	18	0	18	1.000	3.000	1.944	0.802
RAQ48678.1	18	0	18	1.000	3.000	1.889	0.900
RAQ48988.1	18	0	18	1.000	5.000	2.333	1.455
RAQ49094.1	18	0	18	1.000	5.000	2.667	1.188
SMN23020.1	18	0	18	1.000	5.000	2.500	1.425
SMN35232.1	18	0	18	1.000	3.000	1.444	0.784
SMN35255.1	18	0	18	1.000	5.000	2.556	1.247
SMN35259.1	18	0	18	1.000	3.000	1.611	0.778
SPD79481.1	18	0	18	1.000	3.000	1.444	0.784
tr|A0A2N9DMR0|A0A2	18	0	18	1.000	3.000	1.444	0.784
tr|A0A2N9DMR5|A0A2	18	0	18	1.000	4.000	2.111	1.023
tr|H1W8U7|H1W8U7_9	18	0	18	1.000	4.000	1.611	1.145
TVU52697.1	18	0	18	1.000	4.000	2.056	1.211
TVU52963.1	18	0	18	1.000	4.000	1.778	1.114
TVU53417.1	18	0	18	0.000	3.000	1.500	0.924
TVU53586.1	18	0	18	1.000	3.000	1.611	0.778
TVU53692.1	18	0	18	1.000	4.000	1.611	1.145
TVU54284.1	18	0	18	1.000	3.000	1.444	0.784
TVU54640.1	18	0	18	1.000	5.000	1.833	1.295
TVU55119.1	18	0	18	0.000	4.000	1.833	1.200
TVU55427.1	18	0	18	1.000	3.000	1.611	0.778
VIP01172.1	18	0	18	0.000	3.000	1.556	0.856
VIP01439.1	18	0	18	1.000	3.000	1.444	0.784
VIP01757.1	18	0	18	1.000	3.000	1.444	0.784

VIP01864.1	18	0	18	1.000	6.000	2.444	1.977
VIP03226.1	18	0	18	1.000	4.000	2.222	1.060
VIP03488.1	18	0	18	1.000	3.000	1.611	0.778
VIP03920.1	18	0	18	1.000	3.000	1.444	0.784
VTR91003.1	18	0	18	1.000	3.000	1.889	0.900
VTR92413.1	18	0	18	1.000	3.000	1.444	0.784
VTR92436.1	18	0	18	1.000	3.000	2.056	0.802
VTR92544.1	18	0	18	1.000	3.000	1.778	0.943
VTR93808.1	18	0	18	1.000	4.000	2.222	1.114
VTR94017.1	18	0	18	1.000	5.000	2.500	1.339
VTR94710.1	18	0	18	1.000	3.000	1.444	0.784
VTR96031.1	18	0	18	1.000	4.000	2.111	1.023
VTR96211.1	18	0	18	1.000	4.000	2.056	1.211
VTR96478.1	18	0	18	2.000	6.000	3.667	1.283
VTR97771.1	18	0	18	1.000	4.000	2.389	0.979
VTR98805.1	18	0	18	1.000	6.000	1.944	1.514
VTR99319.1	18	0	18	1.000	3.000	1.889	0.900
VTS00916.1	18	0	18	1.000	5.000	2.333	1.188
VTS01204.1	18	0	18	1.000	4.000	1.944	1.056
VTS02576.1	18	0	18	1.000	5.000	2.444	1.580
VTS03217.1	18	0	18	1.000	3.000	1.444	0.784
VTS06586.1	18	0	18	1.000	3.000	2.056	0.802
VTS09788.1	18	0	18	1.000	6.000	3.056	1.765
VTS10391.1	18	0	18	1.000	3.000	1.444	0.784
VTS10439.1	18	0	18	1.000	3.000	2.056	0.802
VTS10532.1	18	0	18	1.000	3.000	2.278	0.575
VTS11136.1	18	0	18	1.000	3.000	1.944	0.873
VTS11826.1	18	0	18	1.000	3.000	1.444	0.784
VTS12076.1	18	0	18	1.000	3.000	1.944	0.873
VTT98366.1	18	0	18	1.000	5.000	2.444	1.580
VTT99252.1	18	0	18	1.000	3.000	1.444	0.784
VTT99839.1	18	0	18	2.000	10.000	5.722	2.845
VTU00757.1	18	0	18	1.000	3.000	1.444	0.784
VTU01064.1	18	0	18	0.000	4.000	2.222	1.309
VTU01302.1	18	0	18	1.000	3.000	1.444	0.784
VTU01377.1	18	0	18	0.000	3.000	1.444	0.856
VTU01600.1	18	0	18	1.000	4.000	2.556	1.042
VTU01960.1	18	0	18	1.000	5.000	1.611	1.195
WP_046320831.1	18	0	18	1.000	6.000	1.611	1.335
WP_046320891.1	18	0	18	1.000	4.000	1.389	0.850
WP_046321251.1	18	0	18	1.000	5.000	1.722	1.127
WP_052741562.1	18	0	18	1.000	3.000	1.278	0.575
WP_084831741.1	18	0	18	1.000	4.000	2.222	1.114
WP_084831749.1	18	0	18	1.000	3.000	1.444	0.784
WP_108614728.1	18	0	18	1.000	3.000	1.444	0.784
WP_108614978.1	18	0	18	1.000	3.000	1.444	0.784
YP_005098423.1	18	0	18	1.000	3.000	2.111	0.758
